# Ancient eukaryotic protein interactions illuminate modern genetic traits and disorders

**DOI:** 10.1101/2024.05.26.595818

**Published:** 2024-05-29

**Authors:** Rachael M. Cox, Ophelia Papoulas, Shirlee Shril, Chanjae Lee, Tynan Gardner, Anna M. Battenhouse, Muyoung Lee, Kevin Drew, Claire D. McWhite, David Yang, Janelle C. Leggere, Dannie Durand, Friedhelm Hildebrandt, John B. Wallingford, Edward M. Marcotte

**Affiliations:** 1 Department of Molecular Biosciences, The University of Texas at Austin, Austin, TX 78712, USA; 2 Division of Nephrology, Department of Pediatrics, Boston Children’s Hospital, Harvard Medical School, Boston, MA 02215, USA; 3 Department of Biological Sciences, University of Illinois at Chicago, Chicago, IL 60607, USA; 4 Lewis-Sigler Institute for Integrative Genomics, Princeton University, Princeton, NJ 08544, USA; 5 Department of Biological Sciences, Carnegie Mellon University, 4400 5th Avenue Pittsburgh, PA 15213, USA

## Abstract

All eukaryotes share a common ancestor from roughly 1.5 – 1.8 billion years ago, a single-celled, swimming microbe known as LECA, the Last Eukaryotic Common Ancestor. Nearly half of the genes in modern eukaryotes were present in LECA, and many current genetic diseases and traits stem from these ancient molecular systems. To better understand these systems, we compared genes across modern organisms and identified a core set of 10,092 shared protein-coding gene families likely present in LECA, a quarter of which are uncharacterized. We then integrated >26,000 mass spectrometry proteomics analyses from 31 species to infer how these proteins interact in higher-order complexes. The resulting interactome describes the biochemical organization of LECA, revealing both known and new assemblies. We analyzed these ancient protein interactions to find new human gene-disease relationships for bone density and congenital birth defects, demonstrating the value of ancestral protein interactions for guiding functional genetics today.

## INTRODUCTION

The last eukaryotic common ancestor (LECA), existing approximately 1.5 to 1.8 billion years ago, was the unicellular ancestor of all extant eukaryotes [1,2]. The molecular makeup of this basal eukaryote may offer insights into the ancient genetic innovations that gave rise to the vast eukaryotic cellular complexity we observe today. As such, understanding the core genetic toolkit of LECA has been a long-standing goal in genetics and evolutionary biology.

Previous phylogenomic analyses are in strong agreement that LECA was highly complex and almost certainly contained most hallmarks of the eukaryotic cell. Synthesis of these studies suggests LECA had at least one nucleus [3,4] with linear chromosomes and centromeres [5]; an interconnected endomembrane system comprised of an endoplasmic reticulum [6], Golgi apparatus [7], vesicle trafficking system [8], and nuclear envelope [9]; a dynamic actin- and tubulin-based cytoskeleton [10] including pseudopodia [11], centrioles [12], and at least one cilium [13]; distinct degradative vesicles such as lysosomes [14] and peroxisomes [15]; and mitochondria capable of both aerobic and anaerobic respiration [2,16].

While there are many partial descriptions of the genetic content of LECA, systematic reconstructions of LECA genes are sparse [17–20]. Moreover, there is as yet no integrated picture of LECA’s proteome or how these proteins interact in higher-order assemblies. Such interactome data would be useful because nearly every significant cellular process appears to rely on assemblies of proteins working together [21], often organized into extended networks. Thus, an interactome for LECA would provide a richer portrait of its genetics and biochemistry than is possible from genomic reconstructions alone. Moreover, since proteins are the primary drivers of molecular phenotype [22], protein interaction networks are valuable tools for inferring protein functions and uncovering genotype-to-phenotype relationships of medical and agricultural interest.

Previous efforts to map eukaryotic protein interactomes systematically have primarily concentrated on opisthokonts (*i.e*., animals and fungi) [23–29] with the exception of a handful of studies in plants (e.g. [30–32]) and protists [33,34]. However, comparing protein networks across more divergent species could provide many new insights not only into evolutionary conservation, but also the divergence of specific biological processes. For example, conserved cellular machinery can be used in different organism-specific contexts to produce distinct organism-specific phenotypes (e.g. the “phenolog hypothesis” [35]).

Here, we sought to reconstruct the LECA protein interaction network and to use this network to illuminate the etiology of human genetic disease. We first derived a conservative estimate of the LECA protein-coding gene set. We then integrated ~26,000 mass spectrometry experiments across 31 eukaryotes spanning ~1.8 billion years of evolution to generate a draft map of protein interactions present in LECA and widely conserved across the eukaryotic tree of life. We then used directed analysis of frogs, mice, and humans to demonstrate that these ancient interactions are enriched for human disease-linked proteins. Thus, the LECA interactome serves as both a guide for better understanding the deep origins of eukaryotic systems and as a framework for identifying new disease associations for human proteins.

## RESULTS AND DISCUSSION

### Inferring the gene content of LECA, the last eukaryotic common ancestor

**Figure 1.**
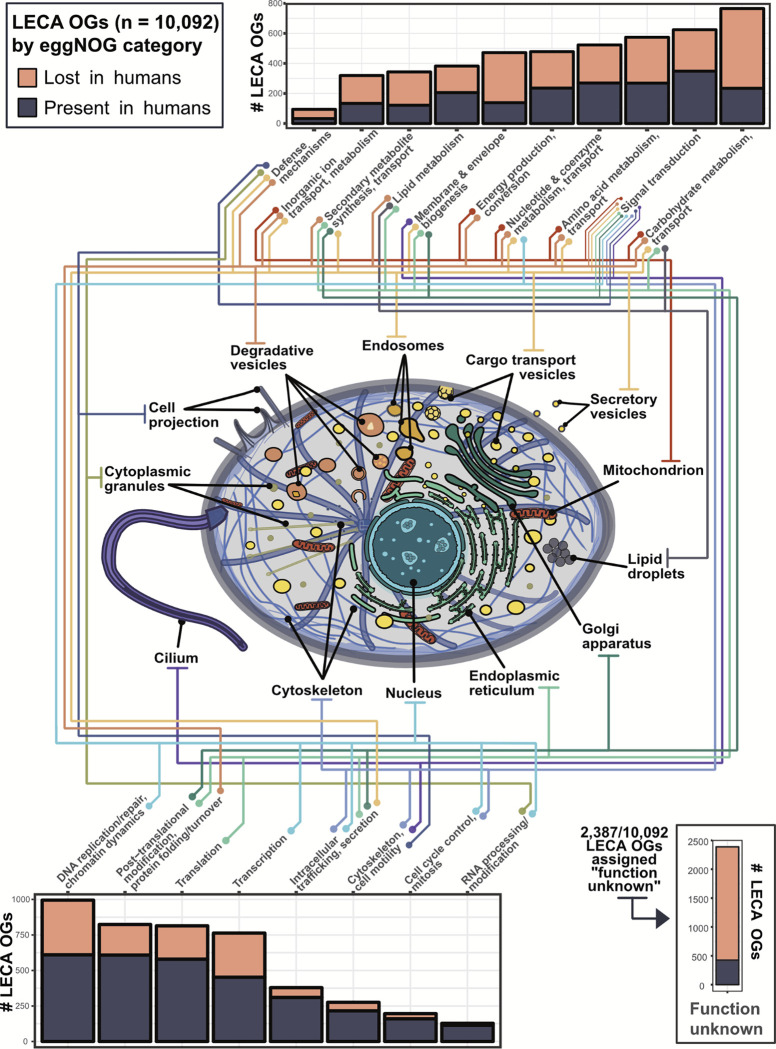
Inferred subcellular organization in LECA, the last eukaryotic common ancestor, based on its estimated gene content. Cell illustration adapted from multiple graphics sourced from SwissBioPics [41].

We first determined a set of 10,092 groups of orthologous genes tracing back to the last eukaryotic common ancestor using Dollo parsimony [36] on reference proteomes from 156 species (see **Methods** and supporting Zenodo data repository). These were defined using the eggNOG algorithm [37] and are referred to hereafter as LECA OrthoGroups (OGs) (Table S1). Within these, we recover genes consistent with the suite of eukaryotic features inferred from ancestral trait reconstructions. We mapped these LECA OGs to known functional annotations [37]. Figure 1 highlights LECA OG functions associated with the nucleus, endoplasmic reticulum, Golgi apparatus, endosomes, digestive vesicles, transport vesicles, secretory vesicles, mitochondria, cilium and an extensive cytoskeleton likely capable of cell projection.

A substantial proportion of functionally annotated LECA OGs are dedicated to DNA replication/repair, transcription, translation, and RNA processing (~25%), underscoring eukaryote-specific innovations related to the segregation of transcription and translation within the nucleus and cytoplasm, respectively [38]. Examples of these innovations are the spliceosome, nuclear envelope, nuclear pore complex, and transport factors such as karyopherins regulated by the Ran-GTP system [9,39]. Another large group of genes (~30%) reflects the expansion and specialization of proteins and pathways related to the compartmentalization of energy production, energy conversion, and metabolism [40]. For example, we recover all V-ATPase subunits, a conserved protein complex responsible for the acidification of lysosomes and peroxisomes. Additionally, we traced mitochondrial-specific genes such as TIM and TOM translocases to LECA, in addition to a large suite of mitochondrial carrier family (MLF) proteins and generalist solute carrier (SLC) family proteins. Continuous MLF- and SLC-mediated transport of myriad metabolites across the mitochondrial membrane enables multiple modes of compartmentalized energy conversion [41], suggesting that LECA had already evolved sophisticated discriminatory pathways necessary for precise partitioning of varied substrates.

LECA also possessed a rich system of endomembranes, with ~15% of LECA OGs associated with membrane biogenesis, intracellular trafficking, and signal transduction. These include endosomal coat proteins such as clathrin, adaptin, and the COPI and COPII vesicle coat complexes which facilitate vesicle budding from the Golgi apparatus and endoplasmic reticulum, respectively [8]. Proteins related to signal transduction underwent a massive expansion at the root of eukaryotes, particularly within GTPases, such as the Ras, Ran, Rho, Rab, Arf, and dynamin superfamilies [42], highlighting the complexity and diversity of early signaling pathways and their relationships with endomembranes. Surprisingly, using eggNOG functional annotations we only found ~300 LECA OGs (~3%) associated with the cytoskeleton and cell motility (*i.e.*, a cilium) while previous studies reported ~500 OGs [43,44].

Finally, the eggNOG algorithm identified 2,387 LECA OGs (~25%) of unknown function (Figure 1, **bottom right**), the majority of which are absent from the human genome. The high proportion of uncharacterized genes and low count of ciliary genes prompted us to attempt to assign function, or at a minimum, subcellular location, to LECA OGs by using annotations for specific extant proteins in these families present in the UniProt database [45] (see **Methods** and Zenodo repository). This allowed us to recover ~200 LECA OGs associated with cytoskeletal and cell motility pathways that were previously classified as “function unknown” by the eggNOG algorithm, bringing the total up to the ~500 expected OGs based on previous literature [43,44]. In sum, we assign tentative UniProt annotations and subcellular compartments to 1,066 of 2,387 LECA OGs originally categorized as “function unknown” by eggNOG.

Overall, slightly less than half of human genes (n = 9,908) map to 4,777 unique LECA OGs, and these differences can be instructive. For example, the human genome appears to have lost a large number of genes associated with carbohydrate metabolism, amino acid metabolism, and membrane biogenesis, including those for isocitrate lyase (ICL) and malate synthase, enzymes of the glyoxylate cycle. Glyoxylate is a highly reactive aldehyde and glyoxylate cycles have been demonstrated in nearly every other branch of life outside of mammals [46,47]. Because of malate synthase loss, human cells must rely on other enzymes to neutralize glyoxylate: (alanine-glyoxylate aminotransferase (AGT) in the peroxisome or glyoxylate reductase (GRHPR) in the cytosol). Defects in either human enzyme produce primary hyperoxaluria, *i.e.*, renal disease caused by the failure to detoxify glyoxylate [48]. This example highlights how comparative evolution informs mechanisms of human disease.

### Mapping the LECA protein interactome

**Figure 2.**
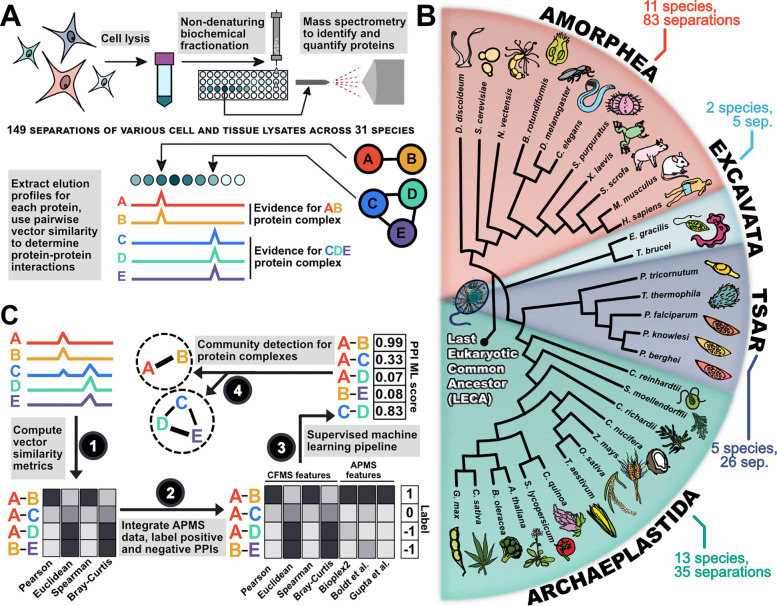
Overview of experimental and computational methods. **(A)** Schematic representation of a co-fractionation mass spectrometry experiment. **(B)** Proteomics data used to construct the LECA interactome included eukaryotes spanning ~1.8 billion years of evolution. Tree structure is based on [26]. Branch lengths are not drawn to scale. **(C)** Schematic overview of the approach for computing protein-protein interaction (PPI) features based on CFMS (1) and APMS (2) datasets, scoring conserved PPIs based on these features (3), and clustering scored PPIs into complexes (4).

We next sought to determine the higher-order organization of the LECA proteome using co-fractionation mass spectrometry (CFMS). CFMS is a high-throughput method for measuring protein-protein interactions (PPIs) *en masse* from virtually any species or cell line without recombinant tags or affinity reagents (Figure 2) [26,49]. CFMS reduces the complexity of a native protein lysate by gently separating protein complexes based on properties such as size, charge, or hydrophobicity, and utilizes the premise that stably interacting proteins will generally co-elute together irrespective of the native separation method used. While results from a single CFMS experiment are insufficient to draw reliable conclusions about specific protein-protein interactions, integrating observations from orthogonal separations from multiple species, cell types, and tissues confers strong statistical power for inferring conserved interactions [27,29,31].

To detect conserved protein interactions, we integrated raw CFMS data from more than 10,000 individual biochemical fractions [26,27,31,33,34] across 31 diverse eukaryotic species spanning ~1.8 billion years of evolution (Figure 2B). In addition to external data sets, we generated new CFMS data for four minimally characterized and phylogenetically diverse unicellular eukaryotes: *Brachionus rotundiformis* (Amorphea, rotifer), *Euglena gracilis* (Excavata, algae), *Phaeodactylum tricornutum* (TSAR, diatom), and *Tetrahymena thermophila* (TSAR, ciliate). Because LECA was ciliated, we expanded our coverage of ciliary proteomes by collecting CFMS data from *Sus scrofa* (Amorphea, pig) tracheal tissue and *Xenopus laevis* (Amorphea, frog) sperm. Experimental details concerning cell types, tissues, developmental stages, and fractionation procedures for each separation can be found either in the **Methods** section or in the PRIDE database (accessions in Table S2).

**Figure 3.**
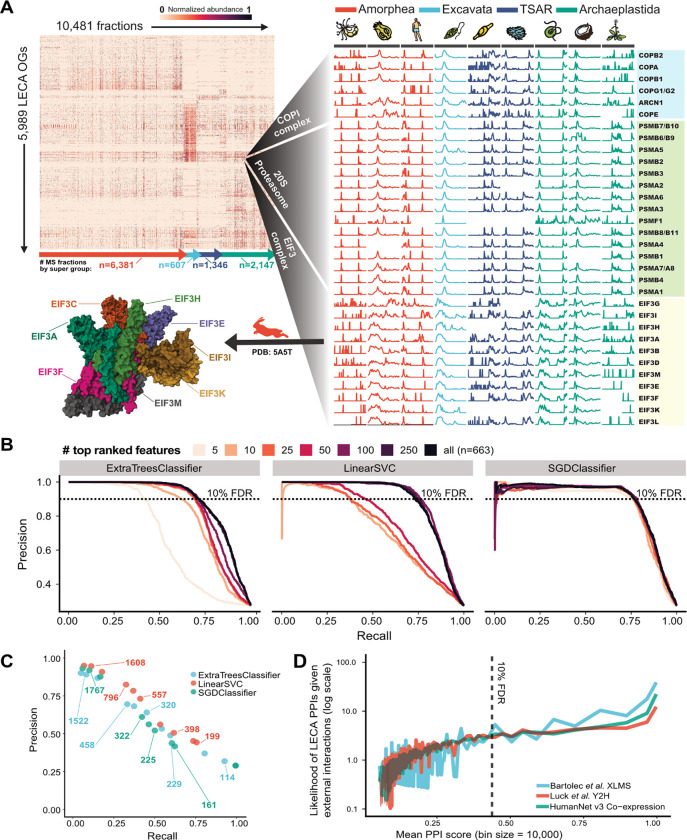
Determining the LECA protein interactome. Co-elution matrix and results of the protein interaction machine learning pipeline. **(A)** Heat map of the filtered elution matrix for 5,989 strongly observed LECA OGs across 10,481 CFMS mass spectrometry fractions (left) and a blow-up of elution vectors for the COPI, 20S proteasome, and eukaryotic initiation factor 3 complexes for a select subset of species (right). **(B)** Precision-recall performance of three classifiers trained with increasingly larger sets of ranked features. **(C)** Precision-recall curves for the reconstruction of known protein complexes defined by a walktrap algorithm, where pairwise PPI scores from each classifier are used as input. Points are labeled with the total number of protein clusters (complexes) constructed at each point in the hierarchy. **(D)** The likelihood that PPIs in our network are present in externally defined protein-protein or mRNA coexpression networks as a function of our model’s PPI score. As PPI scores increase, our model becomes increasingly likely to agree with external studies.

In all, we measured 379,758,411 peptides that were uniquely assigned to 259,732 orthologous groups (or unique proteins not mapping to orthogroups) across the 31 species, 149 separations, and 10,491 fractions (Figure 3A). We further augmented our CFMS data with ~15,000 mass spectrometry proteomics experiments [28] that included affinity purification mass spectrometry (APMS) [50–52], proximity labeling [53,54], and RNA-pulldown data [55]. In total, we incorporated data from 26,297 mass spectrometry experiments. We then filtered this data such that we only retained LECA OGs that were strongly observed, in that the sum total of peptide spectral matches (PSMs) across all 149 fractionations was greater than or equal to 150 PSMs. This resulted in elution profiles for 5,989 well-measured LECA OGs, encompassing approximately 60% of the estimated LECA gene set.

While many complex subunits have co-elution profiles with visually detectable correlation (as for the members of the COPI vesicle coat complex, 20S proteasome, and eukaryotic initiation factor 3 in Figure 3A, **right**), a computational framework is required for systematic identification and to properly control for false positives (Figure 2C). To this end, we employed a supervised machine learning pipeline trained on the data we observed for known protein complexes. We assembled a set of 1,499 known complexes from two databases that record PPIs for a variety of eukaryotic species [56–58] (see **Methods**). We then assessed a number of interaction prediction models generated by three different classification algorithms: extremely randomized trees (“ExtraTreesClassifier”), linear support vector (“LinearSVC”), and stochastic gradient descent (“SGDClassifier”).

We constructed models for each with a variable number of features derived from the mass spectrometry datasets, ranking features on a classifier-by-classifier basis, and evaluated performance by measuring the precision and recall of known complexes withheld from the training data (Figure 3B).

The precision-recall of our initial models were 5–38% recall at 90% precision, similar to previous large-scale protein interaction maps papers [28,29,31]. However, after implementation of a custom data stratification approach (see **Methods**), we observed improvements to performance, with models ranging from 40–90% recall at 90% precision. We suspect that this large jump in performance stemmed from a combination of the large volume of high quality mass spectrometry data being integrated and the data stratification approach, which we found significantly reduced overfitting (see **Methods**).

Using each classifier and its best performing feature set, we identified PPIs with a 10% false discovery rate (FDR) threshold (see Zenodo repository) and clustered the proteins into complexes using an unsupervised community detection “walktrap” algorithm [59], weighting the interactions by their confidence scores. The walktrap procedure determined the “optimal” number of subcommunities to range between 100–400, differing significantly by classifier. These large clusters capture the general organization of eukaryotic cells (e.g., partitions include a large spliceosomal cluster, a large chromosomal maintenance cluster, clusters broadly associated with cilia, etc). In order to obtain more granular protein complexes, we further divided these clusters into increasingly smaller communities to define a hierarchy of protein interactions (Table S3).

**Figure 4.**
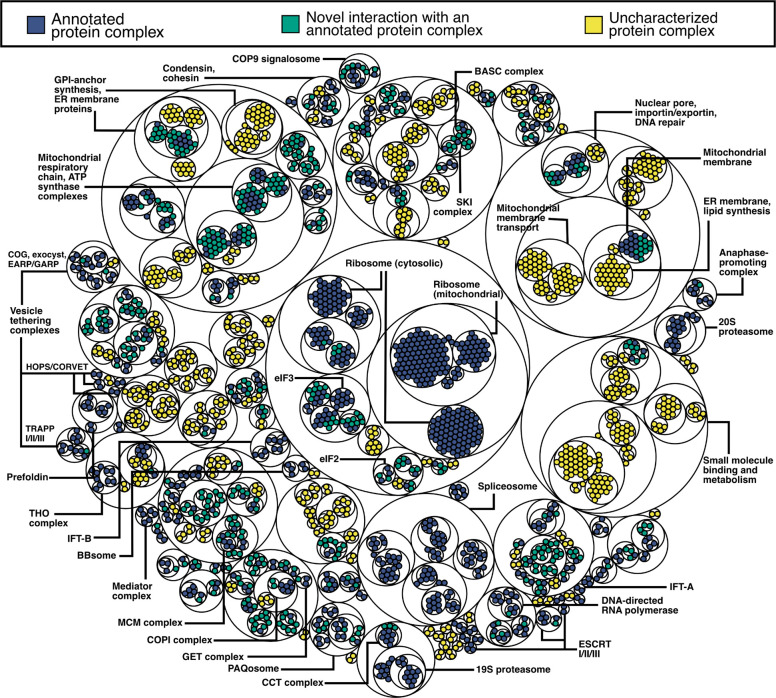
Visualizing hierarchical clustering of protein complexes for a subset of the conserved eukaryotic interactome. The circles of the smallest diameter correspond to individual proteins, where their colors correspond to whether the proteins within each cluster are characterized to interact with each other in the literature (red), whether a novel protein is interacting with a known complex (blue), or whether all the associations within a cluster are uncharacterized (yellow).

As a positive control, we noted that this approach successfully delineated known complexes. For example, a large spliceosome cluster was demarcated into LSM, PrP19 complex, and U4/U6 × U5 tri-snRNP complexes with increased granularity (Table S3). For each classifier, we quantified the performance of the walktrap procedure by computing precision and recall for each cluster at each level of the hierarchy (Figure 3C) and observed a clear tradeoff for increasingly fine-grained subcommunities to show increased precision but decreased recall. Overall, the support vector classifier (Figure 3C, red) netted the highest quality protein complexes and was chosen as our final model. The resulting final LECA complexome consists of the highest confidence 109,466 pairwise interactions between 3,193 unique OGs, hierarchically assembled into 199 (less granular) to 2,014 (more granular) protein complexes, which are portrayed schematically in Figure 4.

We sought to assess how well our final protein interaction model agreed with independent studies that defined interactions using orthogonal approaches. We observe that protein pairs within our highest scoring threshold (≤10% FDR) are significantly more likely than random chance to agree with yeast 2-hybrid (Y2H) [60], mRNA co-expression [61], and cross-linking mass spectrometry (XLMS) [62] interactions (Figure 3D), and performed comparably with a previous interaction map in plants [31].

Finally, current phylogenomic studies hypothesize myriad protein assemblies at the root of eukaryotes [5,8,9,18,63–65]. With an experimentally determined set of conserved LECA PPIs in hand, in the next sections, we examined the extent to which our interactome both recapitulates previously hypothesized and discovers new LECA complexes. We focused on ancient protein assemblies related to intracellular trafficking and cell projection because these are highly relevant to modern human disease [66–68] and highlight below multiple examples where we uncovered previously undescribed interactions.

### Deep conservation and loss of vesicle tethering complexes

**Figure 5.**
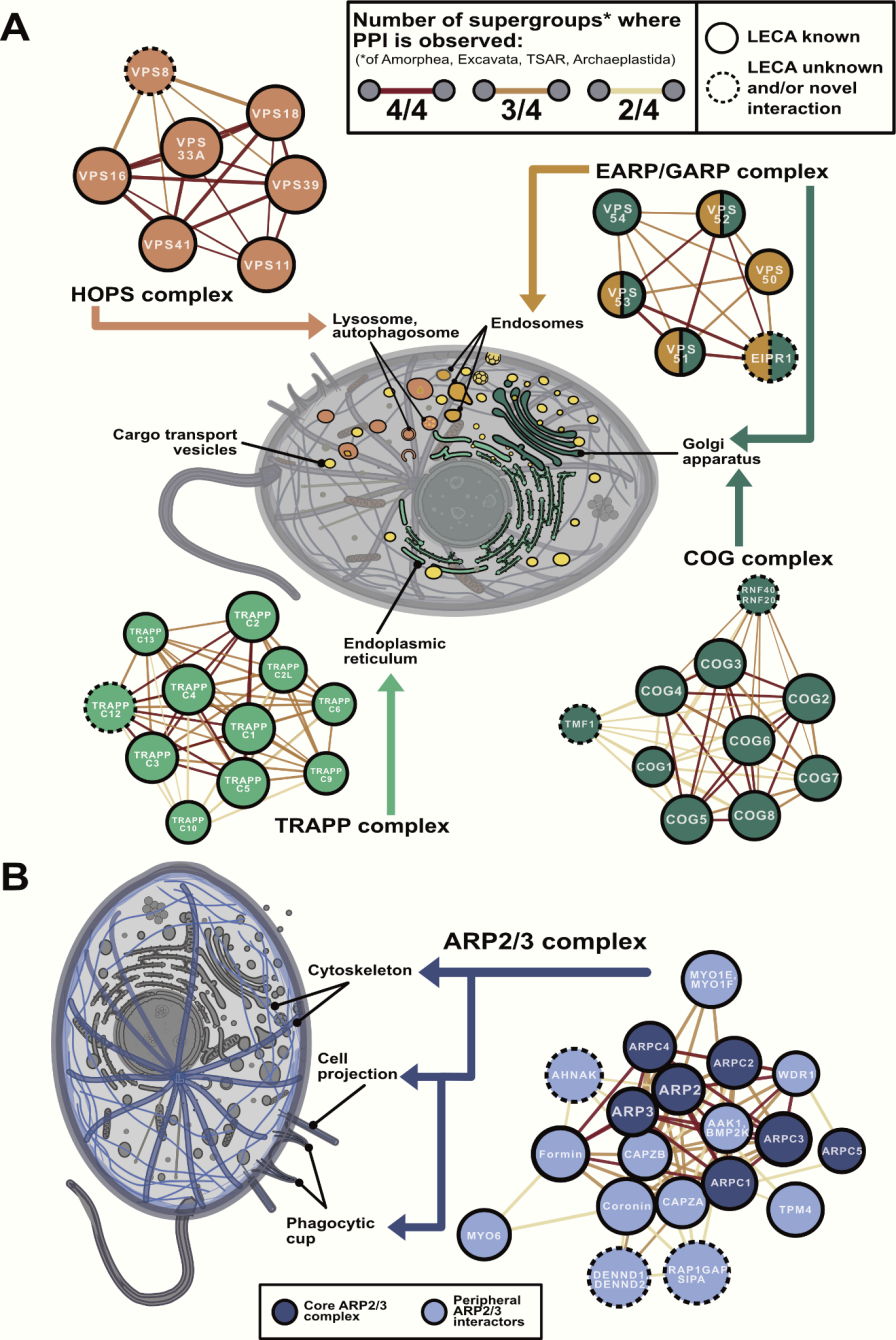
Notable LECA systems related to vesicle tethering and cell projection. **(A)** Node colors for each vesicle tethering complex correspond to their primary subcellular localization: endoplasmic reticulum (light green), Golgi apparatus (dark green), digestive vesicles (orange), or endosomes (yellow). **(B)** Dark and light blue nodes depict core and peripheral cell projection components. In both **(A)** and **(B)**, edges between proteins are colored by the number of eukaryotic supergroups in which the interaction is observed: red for interactions observed in all supergroups considered, orange for interactions observed in three of the four eukaryotic supergroups, and yellow for interactions only observed in half of the supergroups. The four supergroups considered are Amorphea, Excavata, TSAR and Archaeplastida (see Figure 2).

One hallmark of eukaryotic cells is their system of intracellular trafficking by cargo-laden vesicles that bud from one compartment and fuse to another, supported by coat proteins (e.g. clathrin, COPI, and COPII), membrane-anchored SNARE proteins to facilitate membrane fusion [69], and tethering factors ensure target specificity [70]. Many compartment-specific tethering modules are thought to have been present in LECA (as reviewed in [63]), including the ER-associated TRAPP-I, TRAPP-II, and TRAPP-III complexes, the Golgi-associated retrograde protein (GARP), the conserved oligomeric Golgi (COG) complexes, the endosome-associated recycling protein (EARP), and the endolysosomal homotypic fusion and protein sorting (HOPS) complexes. However, the extent to which particular protein interactions are conserved remains an open area of research. In our LECA interactome, we recover all of these core tethering assemblies, along with some unexpected members (Figure 5A).

The GARP and EARP protein complexes are closely related and share three subunits (VPS51, VPS52, VPS53) [71]. Localization is conferred by additional subunits: VPS50 for endosomes or VPS54 for the Golgi apparatus [71]. We observe strongly conserved interactions between all of these subunits, in addition to EIPR1 (EARP and GARP complex-interacting protein 1) (Figure 5A, **top right**). The interaction of EIPR1 with the GARP/EARP complexes was only recently discovered, first in high-throughput screens of human proteins [50,52] and then confirmed in targeted study in human neuroglioma cells [72]. While EIPR1 is speculated to be widely conserved, we find that its interaction with GARP/EARP is indeed ancient and likely traces back to LECA.

In modern eukaryotes the HOPS complex shares four of its six subunits (VPS11, VPS16, VPS18, VPS33) with the related CORVET complex, while the remaining two subunits (VPS39 and VPS41) are unique to HOPS. In yeast, the CORVET subunits direct the fusion of early and recycling endosomes while HOPS directs the fusion of late endosomes, lysosomes, and autophagosomes [73]. Interestingly, we observe conserved interactions between VPS8 (previously thought to be CORVET-specific) and the VPS16, VPS18, VPS39 and VPS41 subunits of the HOPS complex (Figure 5A, **top left**), raising the possibility that a single HOPS-like complex in LECA may have governed the endolysosomal vesicle fusion pathway, with subsequent lineage-specific duplication and specialization of subunits for different compartments.

Analogously, the ER/Golgi-associated TRAPP complex is thought to be composed of five core subunits (TRAPPC1–5) with additional subunits in distinct TRAPP-I (TRAPPC6), TRAPP-II (TRAPPC9, TRAPPC10, TRAPPC13), and TRAPP-III (TRAPPC8) complexes. However, the number and identity of proteins in TRAPP-I/II/III vary significantly by species [74]. In our ancient interactome, we observe strong interactions between each member of the core C1-C5 complex, conserved across all sampled eukaryotic supergroups. Unexpectedly, we find pan-eukaryotic evidence for TRAPPC12 in this core complex, previously thought to be metazoan-specific [75,76]. The remaining interactions are differentially lost in specific eukaryotic lineages, with TRAPPC10 absent in all five sampled TSAR species. Our data, combined with conflicting literature on the exact composition of TRAPP-I/II/III, thus suggests an ancient and flexible core complex where subunits differentially specialize along different eukaryotic branches.

The eight-subunit COG assembly governs retrograde intra-Golgi trafficking and comprises two heterotrimeric subcomplexes (COG2–4 and COG5–7) linked by a COG1-COG8 heterodimer [77]. Our LECA interactome recapitulates this assembly and includes interactions with TMF1 and the ubiquitin ligase complex RNF20-RNF40 (Figure 5A, **bottom right**). TMF1-COG interactions have only been previously observed for metazoan COG2 and COG6 [78], but our data show confident TMF1-COG interactions spanning Amorphea and Archaeplastida, with TMF1 lost in Excavata and TSAR. RNF20-RNF40 is generally described in nuclear roles like histone ubiquitination, transcription regulation, and DNA damage repair [79,80], but has been also linked to the Golgi-associated adapter protein WAC [81], involved in Golgi membrane fusion [82,83]. We see strong conservation of RNF20-RNF40 interactions with COG across Amorphea, TSAR, and Archaeplastida, suggesting that the nuclear-repurposing of this ubiquitin ligase complex could be a recent mammalian innovation.

Thus, the LECA interactome reveals the conservation and specialization of eukaryotic vesicle tethering complexes. We identified unexpected ancient interactions, such as those involving EIPR1 with GARP/EARP, TMF1 and RNF20-RNF40 with COG, and TRAPPC12 with the TRAPP complex, and additionally saw evidence for flexible and lineage-specific adaptations. Given this utility for examining evolutionary conservation and diversification of LECA-associated complexes, we next applied it to shed light on a central question of LECA evolution that is in dispute.

### Primordial origins of cell projection and phagocytosis

While phagocytosis is a trait widely observed across diverse groups of eukaryotes, it is debated whether LECA had the ability to recognize and engulf large particles. Contention stems from arguments concerning how the first eukaryotic common ancestor (FECA) acquired the alpha-proteobacterial precursor of the mitochondrion, *i.e.*, whether FECA was akin to a phagocytosing archaeon or a more “simple” prokaryote that existed in protracted syntrophy with an alpha-proteobacterium [84–86]. Existing phylogenomic investigations into the origins of phagocytosis are conflicting; at least three independent studies conclude phagocytosis probably evolved independently in multiple eukaryotic lineages [87–89], while others argue that the trait was present in LECA and the absence of phagocytosis in certain eukaryotic groups is due to secondary loss as they adapted to new niches [90,91]. Recent investigations into Asgard archaea, the sister group to eukaryotes, reveal a dynamic actin-based cytoskeleton composed of F-actin assemblies, actin-related proteins (Arps), and actin-binding proteins such as profilins and gelsolins capable of modulating eukaryotic actin [92–94], suggesting that FECA may have had phagocytic capacity.

Within our LECA gene set, we find an extensive complement of LECA OGs generally associated both with cell projections (pseudopodia, lamellipodia, filopodia) and with phagocytosis (phagocytic cups and phagosomes) (Figures 1, 5B). Specifically, LECA appears to have had Rho GTPases such as RAC/CDC42, formins, coronins, cofilins, gelsolins, and proteins associated the ARP2/3, ENA/VASP, WASP, SCAR/WAVE, and PI3K complexes. For example, in the LECA interactome, we recovered all seven subunits of the ARP2/3 complex, responsible for the cytoskeletal rearrangement required for cell projection, clustering with other related protein complexes involved in cell protrusion in addition to a number of proteins that are critical for phagocytosis in extant eukaryotic cells (Figure 5B). The core ARP2/3 complex consists of the proteins ARP2, ARP3, ARPC1, ARPC2, ARPC3, ARPC4, and ARPC5. Interestingly, in our data, ARPC5 is the most peripherally associated component in the cluster and has been completely lost in all species sampled within Excavata and TSAR.

Combined with previous evidence [95–97], the presence of these genes in LECA suggests that the ancestral eukaryotic cell was almost certainly capable of pseudopod formation and projection-based motility despite the lack of UniProt annotations in species outside Amorphea (Figure S1). However, because cell projections and phagocytosis share underlying molecular machinery, it is less clear if the presence of these systems necessarily imply a phagocytosing LECA, and more evidence is required to conclude that phagocytosis is an ancestral trait present at the root of eukaryotes. To address this question, we therefore explored the conservation of additional interactions that might shed light on this issue.

Among peripheral interactors of the ARP2/3 complex, we observe CAPZA and CAPZB forming the heterodimeric F-actin capping complex, an essential regulator of actin nucleation that restricts elongation [98], as well as formins and coronins known to promote elongation [95,99]. We also find interactions with WDR1, a promoter of cofilin-mediated actin severing [100] that assists both actin polymerization and depolymerization [101]. Research strongly implicates these systems in Amorphean phagocytosis: Coronins are strongly enriched in phagocytic cups and defects result in impaired phagocytosis in both *Dictyostelium* and mammalian cells [102–104]. Furthermore, we observe protein interaction evidence in at least two major eukaryotic supergroups consistent with the reported roles of AAK1 in receptor mediated endocytosis [105] and unconventional myosin in phagocytosis [106–109]. Taken together, our results strongly support a phagocytosing LECA.

### The LECA interactome reveals a ciliary mechanism for *EFHC2*-associated renal failure

The conservation of protein interactions over billions of years of evolution implies that they are strongly constrained and that their malfunction is likely to be pathogenic. Thus, studying genetic variation through the lens of conserved protein interactions should clarify mechanisms of genetic disease development, tolerance, and resilience. Consequently, we expect the LECA protein interactions to offer direct insights into human disease genetics, and by similar logic, to genotype-phenotype relationships of other modern eukaryotes.

**Figure 6.**
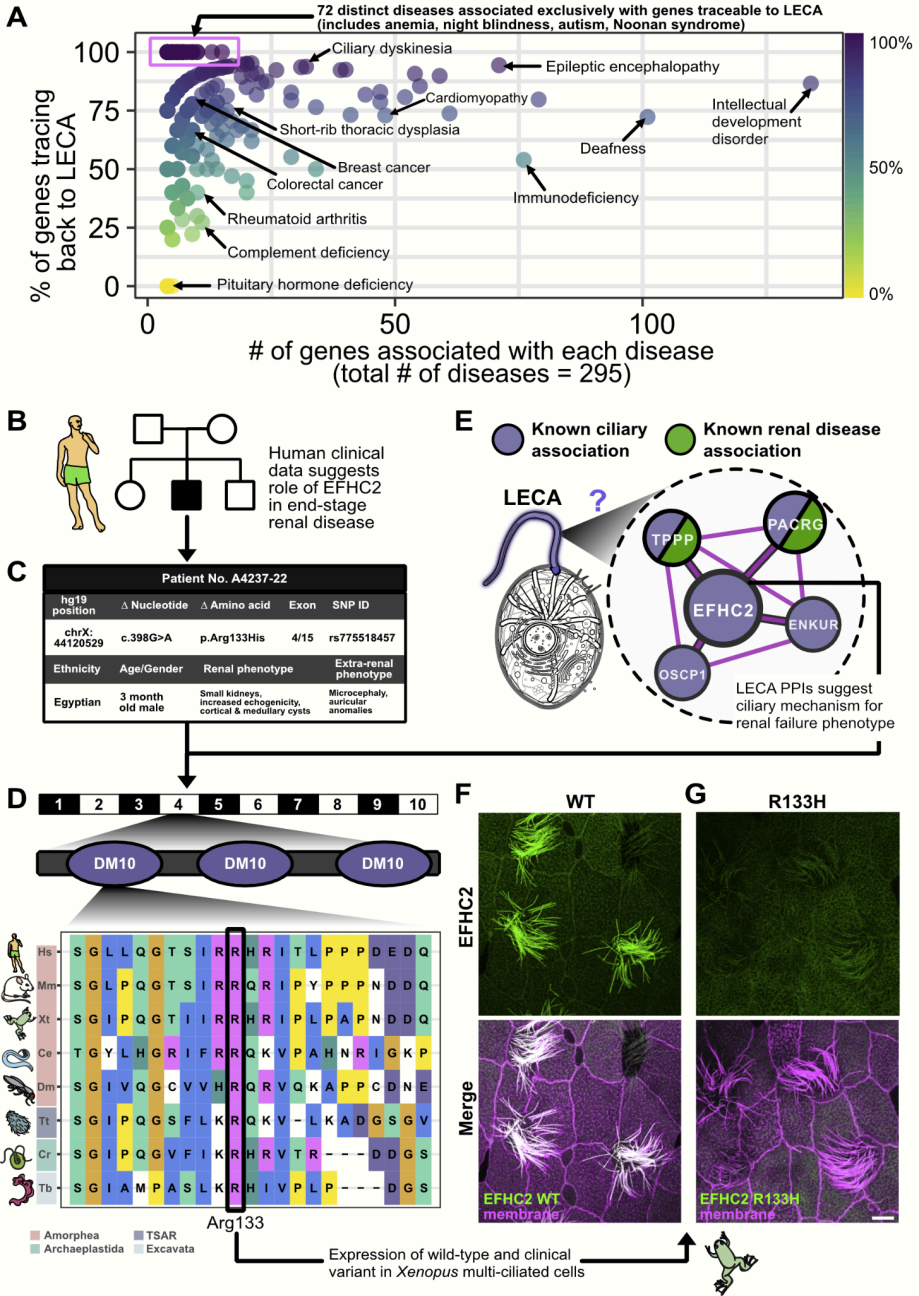
LECA protein interactions suggest mechanisms of genetic disease, as for end stage renal disease gene *EFHC2*, identified by whole exome sequencing and confirmed to have a ciliary etiology. **(A)** Causal genes for human diseases are frequently ancient, as shown by plotting gene-disease relationships obtained from OMIM, with each point representing a unique disease group with an associated number of genes (x-axis) and age, determined as the percentage of genes in LECA OGs (y-axis). **(B)** Pedigree of the index family A4237. Squares represent males, circles females, black shading the affected proband individual A4237–22 included in whole-exome sequencing (WES), and white shading the unaffected parents and siblings. **(C)** Summary of the phenotype and recessive disease-causing R133H *EFHC2* variant identified by WES. **(D)** Location of Arginine 133 in relation to *EFHC2* exon/intron (black/white) structure and DM10 protein domains (purple), and its deep evolutionary conservation. **(E)** EFHC2-containing ciliary complex uncovered in the LECA interactome. **(F)** Localization of GFP-EFHC2 to axonemes in *Xenopus* motile cilia. **(G)** Introduction of the R133H mutation results in loss of ciliary localization of GFP-EFHC2, confirmed by co-labeling with membrane-RFP. Scale bar = 10 μm

Slightly less than half of human genes date back to LECA. Where these conserved genes have been characterized, they have been shown to be responsible for a large and diverse subset of major human diseases, spanning developmental disorders, cancers, chronic respiratory diseases, neurodegenerative conditions, and motor disorders (Figure 6A). For example, of the ~100 human genes known to be associated with deafness, nearly ¾ were present in LECA (Figure 6A). While some human diseases are “new”, evolutionarily-speaking, such as deficiency of the animal-specific pituitary hormone, many other diseases, such as ciliary dyskinesia, arise nearly entirely from genes in LECA OGs. In order to test the utility of our data for illuminating the biology of extant species, we next asked if the LECA interactome could be leveraged to predict human disease mechanisms and novel gene-disease relationships.

Approximately 500 LECA OGs are related to cilia, and among the most common ciliopathies are diseases of the kidney [110]. We identified a male infant with microcephaly, seizures, polycystic kidney disease, and end-stage renal failure, and whole exome sequencing and pedigree analyses revealed a significant hemizygous, X-linked G>A variant in *EFHC2* (rs34729789, 11:44148852:G:A) (Figures 6B–D, S2). Essentially nothing is known of the function of *EFHC2*, though it and its paralog *EFHC1* encode proteins thought to be microtubule inner proteins (MIPs) that function specifically in motile cilia; loss of their orthologues in *Chlamydomonas* or *Tetrahymena* leads to defective ciliary axonemes and/or ciliary beating, but do not disrupt ciliogenesis [111,112]. Because motile cilia are not present in mammalian kidneys, the link between *EFHC2* and this patient’s disease was surprising. We therefore examined our LECA interactome for insights.

We first noted that the patient’s missense variant altered an arginine residue at position 133 to histidine, and this residue is conserved across Archaeplastida, Excavata, TSAR, and Amorphea (Figure 6D). Moreover, EFHC2 was closely and exclusively linked in our LECA interactome to other proteins involved in cilia motility (Figure 6E). We therefore examined the protein’s localization in *Xenopus* multiciliated cells, and found it to be very strongly localized to ciliary axonemes. By contrast, the disease-associated R133H variant failed to localize to cilia (Figure 6F, G), suggesting that defective ciliary localization of this protein contributed to ciliopathic kidney disease in the affected child.

This result then prompted us to ask if other proteins in the cluster, which are also thought to function specifically in motile cilia, might also be implicated in kidney disease. Indeed, previous genomic analyses link both *PACRG* [113] and *TPPP* [114] to chronic kidney disease, with *PACRG* specifically linked to end-stage renal disease [113]. This combination of clinical data, the LECA interactome, and specific hypothesis testing in a vertebrate model organism thus links EFHC2 ciliary function to an end-stage renal disease for which the molecular etiology was previously unknown and underscores the power of our comparative evolution strategy.

### Network propagation for systematic ranking of gene-disease relationships

**Figure 7.**
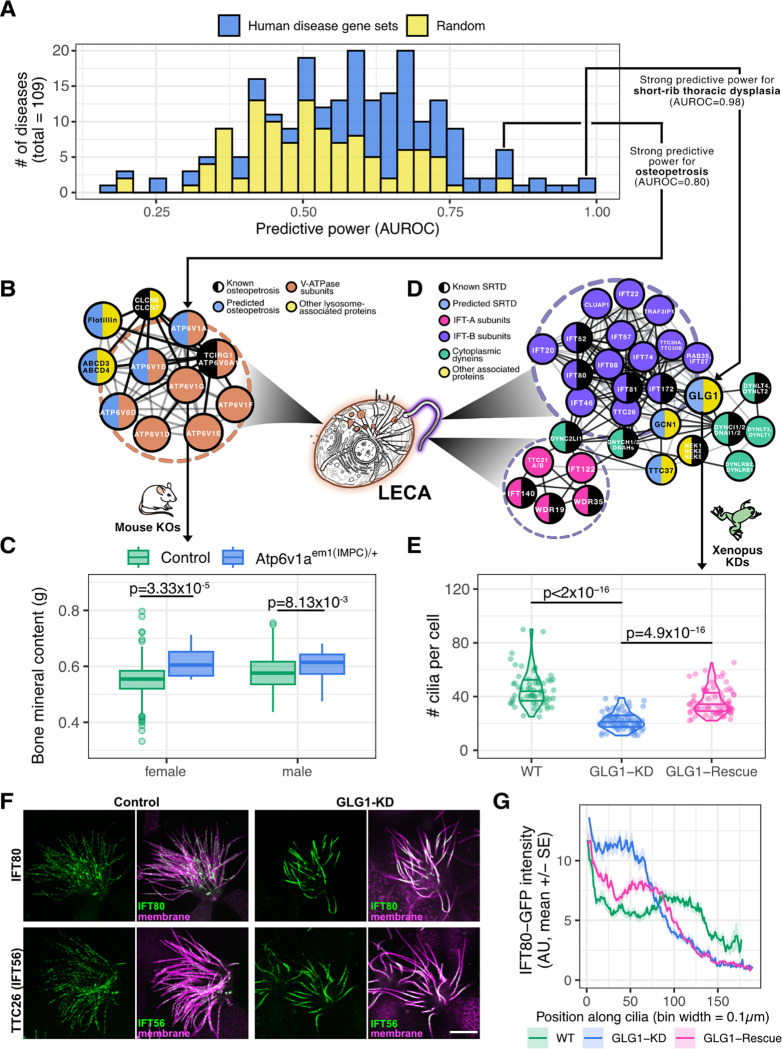
Guilt-by-association in the LECA interactome identifies *ATP6V1A* as causative for osteopetrosis and *GLG1* for short-rib thoracic dysplasia (SRTD). **(A)** Guilt-by-association in the LECA PPI network correctly associates genes to human diseases for roughly a third of the 109 diseases tested, measured as the areas under receiver operating characteristic curves (AUROCs) of leave-one-out cross-validated predictions of known disease genes (light blue) versus random associations (yellow). **(B)** PPI network of genes clinically linked to osteopetrosis (black half-discs; 3 additional genes lie outside this cluster), the highest-ranking new candidates (purple), and their interactions with other V-ATPase subunits that were not indicated for osteopetrosis (orange). **(C)** For the top-scoring gene *ATP6V1A*, the bone mineral content is plotted for knockout (KO) mice with a heterozygous exon deletion in *ATP6V1A* (*n*=8 for each sex, *n*=16 total) compared to healthy control mice (female *n*=834, male *n*=780). Null mice show significantly increased bone density, consistent with the clinical manifestation of osteopetrosis. **(D)** The PPI network of genes clinically linked to SRTD (black half-discs) implicates *GLG1* (yellow) and suggests a ciliary role, based on interactions with intraflagellar trafficking IFT-A (blue) and IFT-B (purple) complexes, cytoplasmic dyneins and dynactins (green), and other interactors (gray). **(E)** Morpholino knockdown (KD) of *GLG1* significantly reduced the number of cilia in *X. laevis* multi-ciliated cells (Bonferroni adjusted t-test *p* < 10^−16^, *n* = 60 control cells, 79 knockdown cells, and 76 rescue cells, 9 embryos per condition over 3 injection replicates) compared to uninjected control animals; rescue by co-injection with a non-targeted *GLG1* allele confirmed specificity. **(F)** In control *Xenopus* multi-ciliated cells, IFT56-GFP and IFT80-GFP, two subunits of IFT-B, are distributed as particles along the ciliary axonemes. However, MO knockdown of *GLG1* leads to the accumulation of IFT-B proteins in the proximal region of axonemes. Scale bar = 10 μm. **(G)** This effect is quantified for IFT80-GFP for 3 cilia per cell for all cells analyzed in panel **(E)**.

We next sought to score potential disease-causative proteins systematically within our conserved interactome on a disease-by-disease basis. To this end, we used cross-validated network guilt-by-association [115] to predict novel gene-disease pairs for 109 unique diseases based on clinically-validated genotype-to-phenotype relationships sourced from the OMIM database [116] (Figure 7A; see **Methods**). We measured the power of our approach as the areas under receiver operating characteristic curves (AUROC), and compared the predictive performance of known disease-associated gene sets versus that of random gene sets. As expected, random predictions have AUROC scores distributed around 0.5 (Figure 7A, **yellow**). In striking contrast, LECA-interactome predictions were skewed to higher scores (Figure 7A, **blue**), and using a conservative AUROC threshold of 0.7, we made strong new disease candidate predictions for almost one-third of the diseases considered (~35 Mendelian disorders). Below, we discuss the prediction and validation in animals of two such novel protein associations.

### Identification and validation of *ATP6V1A* as a novel candidate for osteopetrosis

Our LECA network propagation approach implicated several vacuolar-type H^+^-ATPase (V-ATPase) proteins in the molecular etiology of osteopetrosis (AUROC ~0.8), a disorder in which bones grow abnormally and become overly dense [117] (Figure 7B). Given the key role of V-ATPases in regulating bone homeostasis by acidifying the space between osteoclasts and bone to help dissolution of bone hydroxyapatite [118], one might assume the disruption of many V-ATPase subunits would result in increased bone density. However, only three subunits have so far been implicated in osteopetrosis in humans [116,119] or mice [120]. The remaining V-ATPase subunits instead display a remarkably broad spectrum of disease associations, including cutis laxa (loose skin) and renal tubular acidosis [121], neurodegenerative disease, deafness [122], Zimmermann-Laband syndrome [123], and even osteoporosis (bone loss) [124], highlighting the need to elucidate the discrete molecular functions of specific V-ATPase subunits.

Our LECA network propagation approach gratifyingly made precise predictions, linking three specific subunits (ATP6V1A, ATP6V1B, ATP6V0D) to osteopetrosis (Figure 7B). To confirm these predictions we examined heterozygous CRISPR-Cas9 knockouts of *ATP6V1A* (performed by the KOMP2 high-throughput mouse phenotyping site at the Baylor College of Medicine, see **Methods**) and found these mice showed significantly increased bone mineral content (Figure 7C). The effect size was much stronger in female mice (*p* = 0.00003) than in male mice (*p* = 0.00813), echoing previous observations of sexual dimorphism in the body composition of mammals [125,126]. Despite the obvious lack of bones in the single celled last eukaryotic ancestor, then, our examination of the underlying protein interaction network of that organism nonetheless identified a specific mammalian phenotype with one specific subunit from among a large repertoire of closely related genes.

### Ancient interactions suggest new candidate genes for a lethal human ciliopathy

Our highest scoring disease association (AUROC ~0.98) involved short-rib thoracic dysplasia (SRTD), a severe human ciliopathy characterized by skeletal abnormalities including dysplasia of the axial skeleton that in many cases lethally impairs respiratory function [127]. The disease is strongly associated with proteins involved in Intraflagellar Transport (IFT), the system which moves cargoes into and out of cilia, and this was reflected in our LECA interactome (Figure 7D). Our highest scoring non-IFT protein prediction, however, was the Golgi protein GLG1 [128]. This was an interesting candidate because mouse mutants of GLG1 display defects in rib development similar to SRTD [129], yet the protein has never been implicated in any aspect of ciliary biology.

We therefore explored the function of GLG1 in *Xenopus* multiciliated cells, and found it predominantly localized to the Golgi in MCCs, as expected. We observed no apparent localization at basal bodies or in cilia (not shown). Nonetheless, knockdown of GLG1 resulted in a significant loss of cilia from MCCs, an effect that was specific since it could be rescued by expression of GLG1-FLAG (Figure 7E). To ask if this defect in ciliogenesis was related to IFT, we performed live imaging of GFP fusions to two components of the IFT complex. In normal cells, both markers labeled small punctae in axonemes of *Xenopus* MCCs, consistent with previous imaging of IFT in these cells [130,131]. By contrast, GLG1 knockdown cells displayed large accumulations of IFT proteins within axonemes (Figure 7F, 7G) that resemble those seen previously after disruption of IFT [130,131].

Thus, analysis of the LECA interactome made a single, specific prediction of ciliary function for just one among the large array of Golgi-resident proteins, and that prediction was validated by experiments in *Xenopus*. These data provided new insights into the still obscure link between IFT and the Golgi [132,133] and, moreover, identified a plausible candidate gene for SRTD.

## Conclusions

Studies of ancient protein-protein interactions and their conservation across species offer valuable insights for exploring the genetic underpinnings of contemporary genetic traits and diseases in modern species. In this work, we took an integrated approach to reconstruct the macromolecular assemblies of ancient proteins that, until now, have only been sparsely described. We defined a core set of likely LECA orthogroups, finding that slightly fewer than half of human genes can be traced back to this set. We integrated those data with more than 26,000 mass spectrometry proteomics experiments, capturing hundreds of millions of unique peptide measurements for hundreds of thousands of unique proteins in species sampled from across the tree of eukaryotes. Using these data, we reconstructed a high-quality conserved LECA protein interactome. This interaction network has formed the core of eukaryotic biology for nearly two billion years, and the dataset reveals new insights into both known protein complexes and novel assemblies.

Consistent with our central premise that the most highly-conserved protein assemblies will tend to be most critical for proper cell and organism function, the LECA interactome successfully predicts mechanisms of human disease and novel gene-disease relationships. We specifically presented evidence for a ciliary mechanism in human *EFHC2*-associated renal failure, identified the V-ATPase subunit ATP6V1A in the etiology of mammalian osteopetrosis, and demonstrated a role for the Golgi protein GLG1 in trafficking IFT-A proteins into cilia as a molecular mechanism for short-rib thoracic dysplasia. Given the intrinsic richness of these datasets, we expect this approach should similarly extend to traits and diseases in most other eukaryotic species, while providing insights into the specific molecular mechanisms involved due to being anchored in deeply conserved ancient protein activities.

## Supplementary Material

Supplement 1
