# Supplementary material for: Ancient eukaryotic protein interactions illuminate modern genetic traits and disorders
Source: bioRxiv. 2024 May 29:2024.05.26.595818. Preprint. [Version 1] doi: 10.1101/2024.05.26.595818 (PMC11160598; doi:10.1101/2024.05.26.595818)
Supplement: Supplement 1 [file NIHPP2024.05.26.595818v1-supplement-1.pdf]

bioRxiv preprint doi: https://doi.org/10.1101/2024.05.26.595818; this version posted May 29, 2024. The copyright holder for this preprint (which was not certified by peer review) is the author/funder, who has granted bioRxiv a license to display the preprint in perpetuity. It is made available under aCC-BY 4.0 International license.

SUPPLEMENTAL METHODS

Resources for inferring the LECA gene set

Reference proteomes for 156 species (122 eukaryotes, 7 archaea, 27 bacteria; see Zenodo repository) were downloaded from the UniProt database along with the corresponding reference species tree [134] for the parsimony analysis. This species tree and set of organisms were selected because they span the tree of life and serve as the gold standards curated by the Quest for Orthologs group for benchmarking orthology inference [135]. The species tree was downloaded from SwissTree (https://swisstree.sib.swiss/cgi-bin/swisst). Analysis of UniProt database reviewed proteins annotated with subcellular localizations was performed using the standardized SL accessions (see Zenodo repository), extracted with REST API queries.

Orthology mapping

Protein sequences from each reference FASTA file were searched against the eggNOG 5.0 database [37] and mapped to orthologous groups (OGs) at the rootNOG level (taxonomic level = 1) using eggNOG-mapper v2.0.5 [136] with DIAMOND and a hit cut-off e-value of 10^{-3}. As a result, 89,955 unique OGs spanning 156 species across the tree of life were used as input to the Dollo parsimony analysis. The group of rootNOGs assigned to the LECA node as a result of the parsimony procedure were converted to euNOGs (taxonomic level = 2759) with a set of hierarchical mapping files provided by Dr. Jaime Huerta-Cepas, the author of the eggNOG algorithm, *via* personal correspondence.

Dollo parsimony

Using the Count evolutionary analysis software [137], we implemented a Dollo parsimony approach [36] across 156 organisms to obtain a conservative estimate of the LECA proteome. The Dollo parsimony model relies on the simplifying assumption that gene loss is irreversible, e.g., once a gene is lost it cannot be regained in a lineage. Thus, we determined the ancestral LECA proteome as the set of orthogroups either (a) shared by the respective outgroups (prokaryotes) and at least one of the eukaryotic species or (b) shared by two eukaryotic groups whose last common ancestor was LECA as defined by the gold standard species tree [138]. This approach has previously been shown to be effective at reconstructing likely LECA orthogroups [19].

Investigation of orthologous groups of unknown function

We attempted to assign functions, or, at the minimum, subcellular localizations, to the 25% of uncharacterized (by eggNOG) LECA OGs using the UniProt database. To this end, we downloaded 363,430 proteins from the UniProt database that were (a) “reviewed” status and (b) assigned a standardized subcellular localization (SL) ID for each compartment that we trace back to the last eukaryotic common ancestor (**Figure**

bioRxiv preprint doi: https://doi.org/10.1101/2024.05.26.595818; this version posted May 29, 2024. The copyright holder for this preprint (which was not certified by peer review) is the author/funder, who has granted bioRxiv a license to display the preprint in perpetuity. It is made available under aCC-BY 4.0 International license.

S1A). The total number of proteins assigned to a subcellular compartment varied by four orders of magnitude, where 166,296 proteins were assigned to the cytoplasm at the highest end and 116 proteins were assigned to phagocytic cups at the lowest end (**Figure S1A**). Furthermore, we investigated the diversity and magnitude of eukaryotic and prokaryotic species contributing to these annotations (**Figure S1B**) and observed an underrepresentation of clades outside Amorphea (see tree in **Figure S3** for supergroup organization). For example, the nucleus is considered a distinguishing feature of eukaryotes; UniProt proteins annotated to localize to the nucleus come predominantly from 1,032 distinct Amorphean species, with almost two orders of magnitude fewer such annotations contributed by non-amorphean species, consisting of 152 archaeplastidans, 2 cryptophytes, 21 excavates, and 62 TSAR species.

To quantify what proportion of the LECA gene set is represented in UniProt, we mapped all 363,420 reviewed UniProt proteins mentioned above to eukaryotic orthologous groups ("euNOGs"; NCBI taxonomic identifier = 2759). This resulted in 13,556 unique euNOGs, the percentage of which that trace back to LECA varies significantly by subcellular localization (**Figure S1C**). As an aside, we note that the same euNOG can often be assigned multiple UniProt SL IDs, netting a total 24,628 euNOG-SL mappings.

Of the 2,790 ciliary proteins that map to 478 unique eukaryotic orthologous groups (euNOGs), nearly ~25% of the 299 UniProt ciliary euNOGs that intersect with LECA OGs were originally assigned "unknown function" by the eggNOG functional annotation algorithm–a larger proportion than most of the other eukaryotic compartments described within the UniProt database. Similarly, of 1,317 cytoskeletal euNOGs, ~60% trace back to LECA and 111 of those were assigned the "function unknown" eggNOG category. Thus, the combination of eggNOG and Uniprot annotations provided a reasonable initial annotation set for subsequent analyses.

Challenges and limitations to defining the LECA gene set

Binning proteins into evolutionarily related orthologous groups with respect to the root of the eukaryotic tree nets a "coarse-grained" mapping of the relationships between eukaryotic genes, *i.e.*, we can not rigorously distinguish orthologs from paralogs. With that said, we are still able to draw conclusions about the properties of families of genes rather than the pairwise relationships of individual members; this approach is intuitive and convenient for large-scale systematic studies and broadly supported [139–141]. However, there is considerable disparity between OG assignment algorithms, though eggNOG has been demonstrated to have among the highest accuracies when tested on a benchmark set of manually curated orthologs [19]. In the same study, the eggNOG algorithm was also shown to perform best at detecting distant homology and properly

bioRxiv preprint doi: https://doi.org/10.1101/2024.05.26.595818; this version posted May 29, 2024. The copyright holder for this preprint (which was not certified by peer review) is the author/funder, who has granted bioRxiv a license to display the preprint in perpetuity. It is made available under aCC-BY 4.0 International license.

splitting out-paralogs, making it the best suited algorithm currently available for our goals. Some protein families and eukaryotic lineages with fast rates of evolution (e.g., transcription factors, proteins associated with the innate immune response, and in general plants that are prone to whole genome duplication [142–146]) remain a weakness to the approach. Proteins such as these are likely "under-split" with respect to their associated orthologous groups. Leucine-rich repeat proteins are a salient example: more than 100 human LRR proteins were assigned to KOG0619, an OG we traced back to LECA related to intracellular trafficking and secretion, and this trend persists across nearly all eukaryotes sampled that had proteins assigned to KOG0619. In this way, we are most likely underestimating the size of the distinct LECA gene set.

Additionally, it should be noted that the Dollo parsimony procedure we used to approximate the LECA gene set, which assumes that the probability that a trait emerges more than once is negligible [36], is the simplest form of ancestral state inference. The use of Dollo parsimony is justified and perhaps even preferable [147–149], given that (a) our goal was to determine a binary character state (the presence or absence of genes), (b) we had a consensus reference species tree in hand [134], (c) the target gene set is eukaryotic wherein independent gene losses are common and gains of multiple genes are (relatively) rare, (d) the expected influence of horizontal gene transfer is minimal (estimated to be ~1% of genes or less [150]), and (e) probabilistic ancestral state reconstruction methods, such as phylogenetic birth-death-gain models, are prohibitively slow for a data set of this size. Nonetheless, one flaw in our approach is worth noting: multiple species within Excavata host plastids or plastid-derived genes orthologous to plastid proteins in plants [151–153], even though it is widely accepted that primary plastids share a single origin [154,155] and Archaeplastida is monophyletic [156,157]. If the Archaeplastidan monophyly is to be believed [158,159], the last common ancestor of Excavata independently acquired plastids (violating Dollo's law), resulting in the inflation of our LECA gene set by ~40 plastid-associated OGs. To correct for this error, we manually removed these OGs from consideration during construction of the LECA interactome.

Resources for interactome mapping

Biological samples, mass spectrometry data sets, and software used in this analysis are summarized in **Table S2**. Proteomes for 31 eukaryotic species were sourced as summarized in **Table S4**.

Mass spectrometry

Native protein extraction and fractionation

For lysates described below protease inhibitor cocktail was cOmplete mini EDTA-free (Roche), phosphatase inhibitors were PhosSTOP EASY pack (Roche), and

bioRxiv preprint doi: https://doi.org/10.1101/2024.05.26.595818; this version posted May 29, 2024. The copyright holder for this preprint (which was not certified by peer review) is the author/funder, who has granted bioRxiv a license to display the preprint in perpetuity. It is made available under aCC-BY 4.0 International license.

all steps after addition of lysis buffers were conducted at 4°C or on ice unless otherwise indicated. Native soluble extracts were quantified by DC Protein Assay (BioRad). All protein samples were 0.45 µm filtered (Ultrafree-MC-HV Durapore PVDF, Millipore) prior to chromatography. Chromatography was performed on an HPLC system as in [31] unless otherwise stated.

Brachionus rotundiformis was collected in batches on Filter Mesh 100 Nylon (~65 µm pore) to remove feeder algae prior to flash freezing in liquid nitrogen. Frozen material (3.1 g) was ground to power in a liquid nitrogen-chilled mortar and pestle and resuspended in an equal volume of *Tetrahymena* Lysis Buffer (25 mM Tris pH7.4, 25 mM NaCl, 1 mM EDTA, 10 % glycerol, 0.2% NP40, with 1 mM DTT, 1 mM PMSF, phosphatase inhibitors, and protease inhibitor cocktail added freshly). Cells were disrupted with 10 strokes in a glass dounce fit with a tight pestle. Following centrifugation 3000 x g, 10 minutes to remove debris, the supernatant was clarified twice by centrifugation 20,000 x g 10 minutes. Size Exclusion Chromatography was performed with 2.6 mg extract in a 200 µl sample loop and mobile phase Buffer S (50 mM Tris-HCl pH 7.5, 50 mM NaCl).

Phaeodactylum tricornutum (UTEX 646) grown without silica was briefly washed by pelleting (2000 x g, 10 minutes, 21°C, no brake) and resuspended in 0.5x artificial seawater (UTEX) before collecting (3000 x g, 4°C, slow deceleration) and flash freezing. Frozen material was ground to powder and allowed to thaw before refreezing and regrinding. 1g of powdered material was resuspended in 800 µl Lysis Buffer (50 mM Tris pH 7.5, 150 mM NaCl 5 mM EGTA, 10% glycerol, 1% NP40 with 0.1mM DTT) with phosphatase inhibitors and Plant Specific Protease Inhibitors (Sigma # P9599). Material was frozen and thawed again before sonicating 6 x 10 seconds on, 20 seconds off, 70% duty cycle. Lysis was monitored by microscopy. The extract was incubated on ice with periodic gentle vortexing for 30 minutes prior to clarification twice at 14,000 x g, 10 minutes. Extract was diluted 3-fold with 50 mM NaCl prior to loading 2 mg for SEC separation as above. For separation by mixed bed ion exchange chromatography (Poly CATWAX A, PolyLC Inc.) salt was reduced by 5x dilution with 10 mM Tris pH 7.5, 5% glycerol, 0.01% NaN_3 and proteins were re-concentrated by ultrafiltration (Amicon Ultra 0.5 ml 10,000 MWCO). IEX chromatography was with 1.9 mg in a 250 µl sample loop.

Euglena gracilis (UTEX 753) was washed briefly by centrifugation (1,500 x g, 5 minutes, 21°C) and resuspension in dH_20, before collection by centrifugation and flash freezing. Material was ground as above and 4.7 g was resuspended in Lysis buffer plus both the cOmplete mini EDTA-free protease inhibitors and the Plant-Specific Protease Inhibitors (Roche). Lysate was sonicated 9 x 10 seconds on, 20 seconds off, 60% duty cycle, followed by gentle nutation 30 minutes. Debris was removed by centrifugation

bioRxiv preprint doi: https://doi.org/10.1101/2024.05.26.595818; this version posted May 29, 2024. The copyright holder for this preprint (which was not certified by peer review) is the author/funder, who has granted bioRxiv a license to display the preprint in perpetuity. It is made available under aCC-BY 4.0 International license.

1,500 x g, 10 minutes, and the supernatant was further clarified twice with 14,000 x g, 10 minute spins. Final extract was filtered through a 0.45 µm syringe filter (Durapore PVDF, Millipore) prewashed with dH_20. Extract was diluted 4-fold in Buffer S and 2 mg loaded on a 200 µl sample loop for SEC fractionation.

Two fresh pig tracheas (*Sus scrofa*) were shipped on ice from Sierra for Medical Science arriving within 24 hours of harvest. After removal of fat tissues the trachea were slit lengthwise, chopped crosswise into several pieces, and washed with multiple changes of ice cold PBS pH 7.4 to remove serum and blood cells prior to extraction with 100 ml Ca++ shock buffer as in [160] including protease and phosphatase inhibitors at 0.5x concentration and 0.1 mM PMSF. Cilia were released by vortexing and manual agitation for 10 minutes. Debris was pelleted 500 x g, 2 minutes and floating lipids were removed by aspiration. Cilia were collected by centrifugation 12,000 x g 10 minutes and washed once by resuspension and centrifugation. Ciliary pellets were resuspended in Ca++ shock buffer with 1% NP40 to extract soluble proteins and residual axonemes were removed by centrifugation twice at 12,000 x g 10 minutes. Any floating lipids were removed after each spin. Extract was flash frozen until used. Thawed extract was diluted 2-fold with 10 mM Tris pH 7.5, 5% glycerol, 0.01% NaN_3 and re-clarified 12,000 x g 10 minutes prior to ultrafiltration with 30,000 MWCO Ultracel Amicon Ultra 0.5 ml units to load 1.7 mg in a 250 µl sample loop for IEX chromatography.

Tetrahymena thermophila SB715 were grown and cilia extracts made as in [161] except that deciliation was by pH shock according to [162]. 1.5 mg cilia extract was fractionated by mixed bed IEX and 1.2 mg by SEC with SEC mobile phase Buffer S-C (50 mM Tris-HCl pH 7.4, 50 mM NaCl, 3 mM $MgSO_4$, 0.1 mM EGTA). Deciliated *Tetrahymena* “bodies” were collected by centrifugation 1,700 x g, 5 minutes, washed once by resuspension in Deciliation Medium (10 mM Tris-HCl pH 7.4, 10 mM $CaCl_2$, 50 mM sucrose), collected by centrifugation as before and flash frozen until use. *Tetrahymena* body lysate was prepared by liquid nitrogen grinding frozen material before resuspending in an equal volume *Tetrahymena* Lysis Buffer with 0.1 mM PMSF. Lysis was achieved on ice for 10 minutes by pipetting up and down. Debris was removed by centrifugation 3,000 x g, 10 minutes. Supernatant was clarified and floating lipids removed by sequential centrifugations at 40,000 x g, 10 minutes, 45,000 x g 30 minutes, 130,000 x g 1 hour, and 130,000 x g 45 minutes. Extract was diluted (final NaCl 22 mM) prior to loading 2.2 mg on a 250 µl sample loop for IEX chromatography. The remaining extract was flash frozen and thawed later for SEC chromatography. Extract was clarified 25,000 x g 10 minutes immediately after thawing, and again after dilution in Buffer S-C for loading of 1.4 mg in 200 µl sample loop. For DSSO-crosslinked samples, cilia extract was prepared using the pH shock method as above, but 20 mM HEPES pH7.4 was substituted for the 50 mM Tris of the Cilia Wash Buffer. Extract was

bioRxiv preprint doi: https://doi.org/10.1101/2024.05.26.595818; this version posted May 29, 2024. The copyright holder for this preprint (which was not certified by peer review) is the author/funder, who has granted bioRxiv a license to display the preprint in perpetuity. It is made available under aCC-BY 4.0 International license.

concentrated by ultrafiltration in an Amicon Ultra Ultracel 10k NMWL unit (UFC501096) to load 1.5 mg in a 250 µl sample loop. The final concentration of NP40 was 2.75%. Fractionation on a mixed bed IEX was performed with substitution of 10 mM HEPES pH 7.4 for Tris in the chromatography buffers A and B. For crosslinking DSSO was dissolved freshly in dry DMF to 50 mM and then diluted with 10 mM HEPES pH 7.4 to 10.5 mM before dispensing 25 µl into each 500 µl fraction. To ensure activity of the crosslinker the DSSO solution was prepared in 2 consecutive batches to treat a total of 76 column fractions. Crosslinking proceeded 1 hour at room temperature (~21°C) and was quenched by addition of Tris pH 8.0 to 28 mM.

Xenopus laevis sperm were isolated from dissected testes of five or eight J-strain *Xenopus laevis* males. Testes were perforated with a 25-gauge needle, sperm blown out using MMR (Marc's Modified Ringers). Larger debris was allowed to settle, and liquid transferred to a fresh tube. Sperm were collected by centrifugation 1,500 x g, 10 minutes. Supernatant was discarded and the sperm pellet was lysed by resuspension in an equal volume of Sperm Lysis Buffer (10 mM Tris-HCL pH7.5, 20 mM KCl, 5 mM $MgCl_2$, 5% glycerol, 1% n-Dodecy-ß-D-Maltoside (Anatrace) with 0.5 mM DTT added freshly). Lysate was clarified by centrifugation 14,000 x g, 10 minutes. 1.2 mg was loaded for mixed bed IEX column fractionation (PolyLC Mixed-Bed WAX-WCX, PolyLC Inc. #204CTWX0510), and 3 mg for SEC fractionation (BioSep-SEC-s4000, Phenomenex).

Mus musculus (embryonic stem cells) were grown as described in [163]. Cells were harvested without trypsin by washing in ice cold phosphate buffered saline (PBS), pelleted, and placed on ice. A 250 µl cell pellet was lysed on ice (5 min) by resuspension in 500 µl of Pierce IP Lysis Buffer (25 mM Tris-HCl pH 7.4, 150 mM NaCl, 1 mM EDTA, 1% NP-40 and 5% glycerol; Thermo Fisher) containing 1x protease inhibitor cocktail III (Calbiochem). During the 5 minutes, cells were periodically dounce homogenized with a small-clearance glass pestle (pestle B). Approximately 2 mg of total protein was loaded on either a mixed bed IEX column (PolyLC Mixed-Bed WAX-WCX, PolyLC Inc. #204CTWX0510) or a BioSep-SEC-s4000 gel filtration column (Phenomenex) equilibrated in PBS, pH 7.2. HPLC chromatography was as in [31] and collected fractions were processed as described in [163].

Lysate preparation and chromatographic separation of other species is described in [27,31,161,164].

Data acquisition and processing

All column fractions were reduced, alkylated and digested with trypsin for mass spectrometry by either method 1 or 2 of the protocols in [165]. Spectra were collected

bioRxiv preprint doi: https://doi.org/10.1101/2024.05.26.595818; this version posted May 29, 2024. The copyright holder for this preprint (which was not certified by peer review) is the author/funder, who has granted bioRxiv a license to display the preprint in perpetuity. It is made available under aCC-BY 4.0 International license.

as in [31] on either a Thermo Scientific Orbitrap Fusion Tribrid or an Orbitrap Fusion Lumos Tribrid mass spectrometer except as noted below. Euglena data were collected on a Lumos using CID (35%) and a topspeed 75 minute method as in [31]. Spectra for DSSO crosslinked *Tetrahymena* cilia IEX fractions were collected using a 2 hour DDA MS2-MS3 method as described in [161] but processed for protein identifications in this study using the MSBlender pipeline described below.

Computational analyses

Reference database construction

Protein sequences from each of the 31 reference FASTA files in **Table S4** were compared against the eggNOG 5.0 database [37] and mapped to orthologous groups (OGs) at the euNOG level (taxonomic level = 2759) using eggNOG-mapper v2.0.5 [136] with DIAMOND and a hit cut-off e-value of 10^{-3}. For each species, a reference database was constructed where proteins are binned into their respective OGs such that each FASTA entry represents a bin of proteins or protein family; this was accomplished by concatenating each sequence assigned to an OG interposed with a triple lysine sequence. Since we allow for two missed trypsin cleavages in peptide spectra assignment, this triple lysine sequence ensures that we avoid the misassignment of peptides matching a chimera of two binned sequences. The benefits of this approach are three-fold: (1) defining proteomes in terms of OGs enables cross-species comparisons, (2) OG binning recovers peptide mass spectra that otherwise could not be uniquely assigned to highly sequence-similar proteins, and as a natural extension (3) facilitates proteomic analysis of species with high ploidy, e.g., *X. laevis* (allotetraploid) [164] and *T. aestivum* (allohexaploid) [31].

Peptide mass spectra processing

Matching of mass spectra to peptides was performed with MSGF+, X!Tandem, and Comet-2013.02.0, each run with 10ppm precursor tolerance and allowing for fixed cysteine carbamidomethylation (+57.021464) and optional methionine oxidation (+15.9949). Peptide search results were integrated with MSBlender [166] as described in [27,31] with the exception that high confidence (1% FDR) peptide spectral matches were required from two out of the three peptide identification algorithms. In all, we measured 379,758,411 peptides that were uniquely assigned to 259,732 unique proteins and orthogroups across all fractions. These results were filtered such that we only retain orthogroups that (a) were determined to trace back to the last eukaryotic common ancestor and (b) were strongly observed such that the sum total peptide spectral matches (PSMs) across all fractionations was >= 150.

Feature curation for protein-protein interactions

bioRxiv preprint doi: https://doi.org/10.1101/2024.05.26.595818; this version posted May 29, 2024. The copyright holder for this preprint (which was not certified by peer review) is the author/funder, who has granted bioRxiv a license to display the preprint in perpetuity. It is made available under aCC-BY 4.0 International license.

For each orthogroup found in each MS fractionation for each species sample, an elution vector was constructed by concatenating the peptide spectral counts for each orthogroup in each fraction. Four measures were used to compare all pairwise elution vectors: the Pearson correlation coefficient, Spearman's correlation coefficient, Euclidean distance, Bray-Curtis dissimilarity. These measures were computed as described in [165] and were generated for: (1) vectors for 149 individual fractionations, (2) concatenated vectors that include all samples within the Amorphea eukaryotic supergroup, (3) concatenated vectors that include all samples within the Excavate eukaryotic supergroup, (4) concatenated vectors that include all samples within the TSAR eukaryotic supergroup, (5) concatenated vectors that include all samples within the Archaeplastida eukaryotic supergroup, and (6) concatenated vectors that include all eukaryotic samples, netting 616 CFMS features.

In order to specifically target conserved pan-eukaryotic protein interactions, we required elution vectors for each protein-protein interaction (PPI) to have a minimum Pearson r of 0.3 and be observed in at least two of the four eukaryotic supergroups, *i.e.*, Amorphea, Excavata, TSAR, and/or Archaeplastida (**Figure 2B**). This reduced the size of our input data from 17,895,154 pairwise protein comparisons to a curated set of 4,491,719 highly conserved PPIs. Finally, we integrated the intersection of these conserved PPIs with 47 pairwise features generated from an orthogonal collection of ~15,000 mass spectrometry proteomic experiments [28] that include APMS [50–52], proximity labeling [53,54], and RNA-pulldown data [55] to attain our final PPI feature matrix, resulting in a total of 663 features for each of 4,491,719 highly conserved potential pairwise PPIs.

Assembly of gold standard protein complexes

Gold standard protein interactions were downloaded from the CORUM [56] (http://mips.helmholtz-muenchen.de/corum) and Complex Portal [57,58] (https://www.ebi.ac.uk/complexportal) databases. Both databases include protein-protein interactions for multiple species, spanning multiple mammals in CORUM (human, rat, mouse, cow, pig) and many eukaryotes in Complex Portal (human, rat, mouse, cow, pig, yeast, *Arabidopsis*, worm, fly, chicken, snake, fish, frog, rabbit). Redundant complexes were merged, and (to reduce representational bias) any complex with >30 subunits was removed from the gold standard complex set. Finally, UniProt IDs were matched to euNOG IDs and the gold standard complexes were pruned to only include those in the LECA proteome as determined by the ancestral state reconstruction described above.

Machine learning for protein interactions

bioRxiv preprint doi: https://doi.org/10.1101/2024.05.26.595818; this version posted May 29, 2024. The copyright holder for this preprint (which was not certified by peer review) is the author/funder, who has granted bioRxiv a license to display the preprint in perpetuity. It is made available under aCC-BY 4.0 International license.

All gold standard PPIs observed in our filtered data set were labeled as positive interactions. Negative interactions were defined as interactions between proteins in different gold standard complexes (e.g., given two gold standard heterotrimers A-B-C and X-Y-Z, data corresponding to an A-X protein pair would be labeled as a negative interaction). To mitigate class imbalance, the total number of negative labels was limited to 3X the observed number of positive PPIs in our data, resulting in 6,629 total positive PPI and 19,887 total negative PPI labels in our feature matrix.

Positive PPIs that participate in multiple complexes are a potential source of representation bias in the truth set, which can lead to under or overfitting during model training. To overcome this, we implemented a data stratification approach (**Figure S4**). First, all gold standard protein complexes are given a unique numeric ID. All protein pairs within a complex inherit that ID. Negative PPIs receive group labels by randomly sampling the distribution of positive PPI group IDs with replacement. If a protein pair participates in >1 complex, that pair will be labeled with a list of IDs. These ID lists represent networks of overlapping complexes. We implemented transitive closure of the networks by recursively merging ID lists that overlap with each other, netting a fully stratified "supergroup" label for each gold standard PPI in the data set.

A group-based split method (scikit-learn's "GroupShuffleSplit" class) was used to generate 5 sets of test and training data, where 75% of the labeled data was used for training and 25% for testing. Each of the 5 training sets were used as input into TPOT [167], an automated machine learning pipeline built on top of scikit-learn, to find the best classification method, pre-processing steps, and parameters for our data. While TPOT generates an internal cross-validation score to evaluate the performance of different models, the pipeline is agnostic to strata within the training set and is thus subject to overfitting. In each instance, the 25% hold-out set was used as a true test of the optimized models produced by TPOT. These results are reported in **Table S5**. In the majority of cases, TPOT reports the extremely randomized trees (ExtraTrees) algorithm with slightly varying parameters and pre-processing steps as the best model for our data. However, both a stochastic gradient descent and linear support vector classification (SGDClassifier and LinearSVC, respectively) pipeline scored comparably to the ExtraTrees method, so we moved forward with feature selection and model assessment for those pipelines as well.

To further reduce the risk of overfitting, recursive feature elimination with cross-validation (RFECV) was used to obtain feature importances and an optimal feature set for each of the chosen models (see Zenodo data repository). Feature importance was evaluated using either the Gini index (in the case of ExtraTrees) or the absolute value of the coefficients of the linear model (in the case of LinearSVC and

bioRxiv preprint doi: https://doi.org/10.1101/2024.05.26.595818; this version posted May 29, 2024. The copyright holder for this preprint (which was not certified by peer review) is the author/funder, who has granted bioRxiv a license to display the preprint in perpetuity. It is made available under aCC-BY 4.0 International license.

SGDClassifier). While RFECV generates an internal cross-validated test score to determine an optimal feature set, the module is agnostic to stratified data and is thus subject to sampling bias. Again, we employed a custom group-based split approach (scikit-learn's "GroupKFold" class, illustrated in **Figure S5**) to generate 5 sets of test and training data. Since GroupKFold generates test/train splits such that every protein complex supergroup is included in the test data at least once, this allows us to use hold-out test sets to gauge bias in the "best" feature sets output by RFECV while also evaluating feature importance stability.

RFECV determines an optimal feature set following these steps: (1) the input training data is used to fit a given model, resulting in either a Gini index value or coefficient for each feature in the data set; (2) the least important feature(s) are recursively removed and the mean test accuracy is computed using cross-validation across the input training set, (3) the optimal number of features is determined such that mean test accuracy is maximized. Then, we use the holdout test set to evaluate the true performance of the final model as output by RFECV method. True feature importance was assessed by aggregating the RFECV results of each split, and, for each feature, counting the number of times it appears in the "optimal" set output and computing the mean and relative standard deviation of its Gini index/coefficient. The "best" features are those that maximize the number of appearances in the final "optimal" set across GroupKFolds splits, maximize the absolute value of the Gini index/coefficient and minimize relative standard deviation (*i.e.*, low RSD indicates the feature importance is stable across GroupKFold train/test splits).

We measured precision and recall for each model using the top 5, 10, 25, 50, 100, and 250 highest ranked features (determined on a model-by-model basis, in other words, the "top" features for the LinearSVC classifier are different than the "top" features for the ExtraTreesClassifier) and all 663 features (**Figure S6A**), choosing the feature set that had the highest number of PPIs and unique proteins within a 10% FDR threshold (**Figure S6B,C**).

Community detection of protein complexes

Communities of interacting proteins were identified using the walktrap algorithm *via* the igraph library interface in Python. Briefly, the walktrap algorithm detects community structure by executing a user-defined number of random walks from each vertex in a graph. Random walks tend to become "trapped" in strongly connected sub-communities of the graph [59], a behavior that we reinforce by weighting graph edges with the probability scores output by our three PPI models (LinearSVC, ExtraTrees, and SGDClassifier). Then, individual protein complex clusters are partitioned such that the modularity of the network is maximized [168], netting an

bioRxiv preprint doi: https://doi.org/10.1101/2024.05.26.595818; this version posted May 29, 2024. The copyright holder for this preprint (which was not certified by peer review) is the author/funder, who has granted bioRxiv a license to display the preprint in perpetuity. It is made available under aCC-BY 4.0 International license.

"optimal" number of protein complex communities given the input graph structure. The walktrap algorithm performed best with the PPI scored output by the LinearSVC model, and was thus chosen as our final interactome. The features used in the final model are reported in the Zenodo repository.

Validation of the protein interaction model with external data

External interaction datasets were sourced from [60–62]. The likelihood of LECA PPIs (I_L) agreeing with external interaction networks (I_E) was calculated with a formula analogous to an odds ratio, described by the equations below.

$$A = I_E \cap I_L \quad \text{Eq. 1}$$
$$B = I_E \setminus I_L \quad \text{Eq. 2}$$
$$C = I_L \setminus I_E \quad \text{Eq. 3}$$
$$D = I \notin (I_E \cup I_L) \quad \text{Eq. 4}$$

$$A + B + C + D = \frac{N_i(N_i - 1)}{2} \quad \text{Eq. 5}$$

		LECA interaction (I_L) +	LECA interaction (I_L) −
External interaction (I_E)	+	***A***	***B***
External interaction (I_E)	−	***C***	***D***

$$\frac{P(I_{L+} | I_{E+})}{P(I_{L+} | I_{E-})} = \frac{A/(A+B)}{C/(C+D)} = \frac{A(C+D)}{C(A+B)} \quad \text{Eq.6}$$

Challenges and limitation to mapping the LECA protein interaction set

It is important to note that the data used in this study strongly favors humans and mammals in general. Most of the CFMS and APMS experiments in this study are sourced from humans. Of the 25,000 experiments included in this study, approximately 18,000 are derived from human cells. Gold standard protein complexes obtained from the CORUM database are exclusively mammalian, which motivated us to also incorporate gold standard PPIs from the ComplexPortal database. However, though ComplexPortal is more diverse and includes *Arabidopsis* assemblies, the majority of the data is still Amorphean. We took a number of steps to ensure we target pan-eukaryotic proteins and protein interactions; for example, we filtered the MS data to only include proteins that we trace back to LECA as well as require every PPI be observed and reasonably correlated by CFMS in at least 2 of the 4 eukaryotic supergroups (as defined in **Figure 2B**) prior to entry into the machine learning pipeline.

Co-fractionation mass spectrometry identifies stable complexes that survive biochemical fractionation. Stochastic sampling across a large number of species and

bioRxiv preprint doi: https://doi.org/10.1101/2024.05.26.595818; this version posted May 29, 2024. The copyright holder for this preprint (which was not certified by peer review) is the author/funder, who has granted bioRxiv a license to display the preprint in perpetuity. It is made available under aCC-BY 4.0 International license.

fractionations allows us to recover some transient interactions. CFMS is also biased towards abundant and soluble proteins, though we typically employ detergents to improve coverage of membrane proteins. We measure approximately 60% of the 10,092 OGs we trace to LECA (probably due to the above biases) and high precision PPIs for half of these, indicating a high false negative rate. Inclusion of the APMS data sets increases the power of our model for PPI detection but only for systems conserved within Amorphea. Binning proteins into evolutionarily related protein families (orthogroups) prior to peptide identification and assignment results in loss of resolution for different isoforms and some paralogs. For the most part, we are able to draw conclusions about the properties of families of genes rather than individual members. In some cases, we can retroactively disentangle which variant(s) or paralog(s) participate in a specific interaction by examining the specific peptides identified by mass spectrometry.

Resources for disease analyses

We downloaded 17,019 gene-disease relationships from the Online Mendelian Inheritance in Man database (omim.org) [116].

Curation of a gene-disease data set

Ensembl accessions from the OMIM data set were first mapped to human UniProt identifiers and then subsequently matched with their corresponding eggNOG orthologous groups (OGs) at the root of eukaryotes (NCBI taxonomy level = 2759). Gene-disease assignments in the raw OMIM data did not follow a standardized schema and required a combination of programmatic and manual cleaning. For example, AKT1, PTEN, KLLN, and SEC23B are respectively assigned to Cowden syndrome 6, Cowden syndrome 1, Cowden syndrome 4, and Cowden syndrome 7 in the original data set. After cleaning, these genes are grouped under a common “Cowden syndrome” label. We filtered the data set to contain only LECA OGs, netting 5,761 genotype-to-phenotype associations for the network propagation of gene-disease relationships in the conserved eukaryotic interactome. In total, we curated 1,683 unique disease labels for 2,262 highly conserved human genes.

Network propagation

We used a cross-validated network propagation approach to systematically assign disease predictions to proteins in the interactome. If a protein in the network is known to be associated with a disease, then each connecting node receives the score of the connecting edge. This process is repeated for each unique disease label in our OMIM data set given that the disease has at least five mapped associations with genes that also map to an orthologous group in the last eukaryotic common ancestor. To assess the quality of this propagation for each disease, we iteratively leave out true

bioRxiv preprint doi: https://doi.org/10.1101/2024.05.26.595818; this version posted May 29, 2024. The copyright holder for this preprint (which was not certified by peer review) is the author/funder, who has granted bioRxiv a license to display the preprint in perpetuity. It is made available under aCC-BY 4.0 International license.

positive nodes and query how well the propagation recapitulates known gene-disease relationships (*i.e.*, leave-one-out cross validation). We calculate true and false positive rates to construct a receiver operating characteristic (ROC) curve as a function of propagated score. Then, we use the area under the ROC curve (AUROC) as a measure of performance. Additionally, for each disease, we repeat propagation from randomly selected nodes from the network to evaluate the statistical strength of the gene-disease network versus randomly assigned gene-disease relationships.

Experimental analyses of candidate disease genes

Genetic knock outs in Mus musculus

We sourced *Atp6v1a* knockout data (with permission) from the Knockout Mouse Program (KOMP2) sited at the Baylor College of Medicine, which resides under the umbrella of the International Mouse Phenotyping Consortium (IMPC). The IMPC aims to systematically phenotype mice that are homozygous for a single-gene knockout or heterozygous when homozygotes are lethal or sub-viable [169]. Within the IMPC, KOMP2 production centers use the high-throughput and rigorously standardized IMPReSS pipeline to generate and phenotype single-gene knockout mice. Methods for generating single-gene null alleles are described in [170] and phenotype data collection procedures can be accessed in detail at https://www.mousephenotype.org/impress/index.

Atp6v1a mutant alleles were generated by KOMP using CRISPR/Cas9 to introduce a critical exon deletion in a murine C57BL/6N background. Phenotypes were measured from postnatal mice following the embryonic and early adult IMPC pipelines. In the case of *Atp6v1a*, homozygous knockouts resulted in complete penetrance of pre-weaning lethality. As a result, heterozygous knockouts were generated for 8 female mice and 8 male mice. At 14 weeks, bone mineral content and density was measured for each *Atp6v1a*$^{em1(IMPC)Bay/+}$ mutant using dual-energy X-ray absorptiometry (DEXA). The IMPC uses the PhenStat R package to identify abnormal phenotypes from high-throughput pipelines [171]; for the *Atp6v1a*$^{em1(IMPC)Bay/+}$ mutants, a linear mixed model factoring in the effects of sex and body weight was implemented in PhenStat to assess significance of differential bone mineral content.

EFHC2 patient genetics

Individual A4237-22 was a male of Egyptian origin who was diagnosed with small kidneys, increased echogenicity, cortical and medullary cysts, and microcephaly. To identify a potential genetic cause for the individual's phenotype, authors S.S. and F.H. performed whole exome sequencing (WES) analysis on individual A4237-22. Given the parents' unaffected status regarding their renal phenotype, a recessive mode of inheritance was hypothesized.

bioRxiv preprint doi: https://doi.org/10.1101/2024.05.26.595818; this version posted May 29, 2024. The copyright holder for this preprint (which was not certified by peer review) is the author/funder, who has granted bioRxiv a license to display the preprint in perpetuity. It is made available under aCC-BY 4.0 International license.

Homozygosity mapping revealed only 4.4 Mb of homozygosity, confirming the non-consanguinity of the parents (**Figure S2A**). We detected a hemizygous X-linked missense variant in A4237-22 (c.398G>A; p.Arg133His) (**Figure 6D, S2B**). The variant has not been reported as homozygously or hemizygously in the gnomAD database in 166,211 control individuals. The p.Arg133His amino acid resides in the DM10 domain (**Figure 6E**).

The patient's DNA was also screened for potentially deleterious variants in all genes known to cause kidney disease without results.

Protein localization and knockdown experiments in Xenopus laevis

Xenopus embryo manipulations were performed as in [172–174]. Briefly, female adult *Xenopus* were ovulated by injection of hCG (human chorionic gonadotropin). In vitro fertilization was carried out by homogenizing a small fraction of a testis in 1X Marc's Modified Ringer's (MMR). Embryos were dejellied in 1/3X MMR with 2.5% cysteine (pH 7.8) at the two-cell stage. For microinjections, embryos were placed in a 2% Ficoll and 1/3X MMR solution, injected with mRNA using forceps and an Oxford universal micromanipulator, and washed with 1/3X MMR after 2 hours.

The full length sequences of *Xenopus EFHC2* and *GLG1* were downloaded from Xenbase [175]. The DNAs corresponding to the open reading frames (ORFs) of *EFHC2* and *GLG1* were amplified from *Xenopus* cDNA and were cloned into a pCS10R MCC vector containing an N-terminal GFP or a C-terminal FLAG tag driven by an MCC specific alpha tubulin promoter, respectively. The pCS10R MCC *GFP-EFHC2* R133H construct was generated by site-directed mutagenesis (NEB, #E0554S) from pCS10R MCC *GFP-EFHC2*. Capped mRNAs were synthesized using the mMESSAGE mMACHINE SP6 transcription kit (Invitrogen Ambion, #AM1340). A morpholino antisense oligonucleotide (MO) against *GLG1* was designed to block translation (GeneTools). The MO sequence is 5'-CCATCTTGGGAAGTGCTAGTCAAG-3'.

mRNA and MO were injected into two ventral blastomeres of 4-cell stage Xenopus embryos in 2% Ficoll (w/v) in 1/3 X MMR and the injected doses of mRNAs or MO per cell are as follows: *GFP-EFHC2* and *GFP-EFHC2* R133H (78 pg), *GFP-IFT56* and *GFP-IFT80* (100 pg) [131], membraneRFP(50 pg), *GLG1-FLAG* for rescue experiment (700 pg), and *GLG1* MO (30 ng) for the knockdown experiment. Live images were captured at stage 23 or stage 25 with LSM700 inverted confocal microscope (Carl Zeiss) with a Plan-APOCHROMAT 63×/1.4 oil immersion objective or Nikon eclipse Ti confocal microscope with 60×/1.4 oil immersion objective. Imaging analysis was

bioRxiv preprint doi: https://doi.org/10.1101/2024.05.26.595818; this version posted May 29, 2024. The copyright holder for this preprint (which was not certified by peer review) is the author/funder, who has granted bioRxiv a license to display the preprint in perpetuity. It is made available under aCC-BY 4.0 International license.

performed using Fiji. Bonferroni-adjusted *p*-values were calculated in R using the base stats package.

bioRxiv preprint doi: https://doi.org/10.1101/2024.05.26.595818; this version posted May 29, 2024. The copyright holder for this preprint (which was not certified by peer review) is the author/funder, who has granted bioRxiv a license to display the preprint in perpetuity. It is made available under aCC-BY 4.0 International license.

SUPPLEMENTAL FIGURES AND LEGENDS

Figure S1. Phylogenetic analysis of the reviewed UniProt database by subcellular localization. Limitations to available annotations are evident in an analysis of the UniProt protein database across species, where reviewed proteins have assigned subcellular localizations likely present in the last eukaryotic common ancestor. **(A)** Light gray, total number of reviewed UniProt proteins by UniProt SL term; dark gray, total number of unique eukaryotic OGs assigned to UniProt proteins by UniProt SL term. **(B)** Phylogenetic representation of the proteins sourced from UniProt by UniProt SL term. **(C)** The percentage of eukaryotic orthologous groups (euNOGs) that trace back to LECA by UniProt SL term.

Figure S2. **Homozygosity mapping and verification of EFHC2 mutation in individual A4237-22. (A)** Homozygosity mapping depicts a homozygosity of 4.4 Mb and confirms the reported non-consanguinity of the parents. **(B)** Chromatograms obtained by direct sequencing of PCR products reveal a homozygous substitution of C for T in exon 4 of the *EFHC2* gene in A4237-22.

Figure S3. Reference species tree illustration generated by the Interactive Tree of Life [96] for most of the Quest for Orthologs benchmark species (147/156) used in the Dollo parsimony analysis. Branch lengths are not to scale. Major supergroups are highlighted across the tree. Prokaryotic groups include Bacteria (gray) and Archaea (yellow). Eukaryotic groups include Excavata (light blue), Archaeplastida (green), TSAR (purple), and Amorphea (red).

Figure S4. Illustration of transitive closure for grouping gold standard protein complexes into supergroups.

Figure S5. Illustration of group-based k-fold (in this example, k=3) cross-validation for protein-protein interactions.

Figure S6. Model selection and optimization. (A) Precision-recall curves for three different algorithms, varying the number of "top" most important features used as input (duplicate panel to Figure 3B). Feature importance is defined per algorithm, ranked by either the absolute value of coefficients for linear models (LinearSVC, SGDClassifier) or the Gini index (ExtraTreesClassifier). (B) The number of pairwise protein-protein interactions (PPIs) within a 10% FDR threshold for each model. Black stars (★) denote the final models used as input to a community detection algorithm to define protein complexes. (C) The number of unique proteins that have at least one interaction scored within a 10% FDR threshold for each model.

bioRxiv preprint doi: https://doi.org/10.1101/2024.05.26.595818; this version posted May 29, 2024. The copyright holder for this preprint (which was not certified by peer review) is the author/funder, who has granted bioRxiv a license to display the preprint in perpetuity. It is made available under aCC-BY 4.0 International license.

Figure S1.

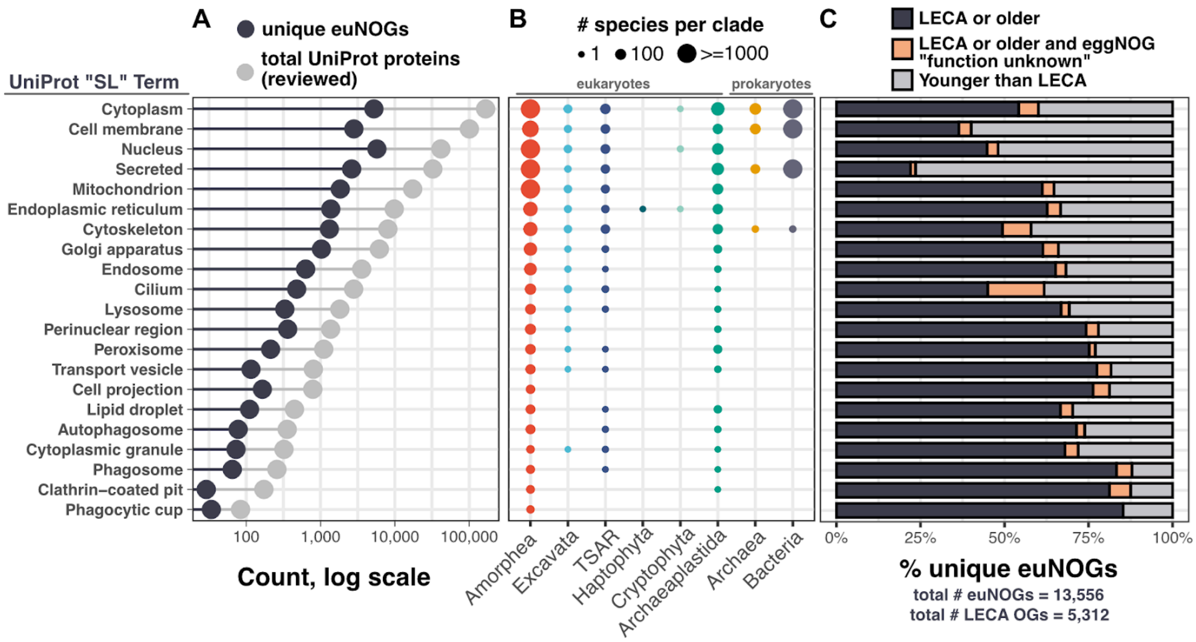

bioRxiv preprint doi: https://doi.org/10.1101/2024.05.26.595818; this version posted May 29, 2024. The copyright holder for this preprint (which was not certified by peer review) is the author/funder, who has granted bioRxiv a license to display the preprint in perpetuity. It is made available under aCC-BY 4.0 International license.

Figure S2.

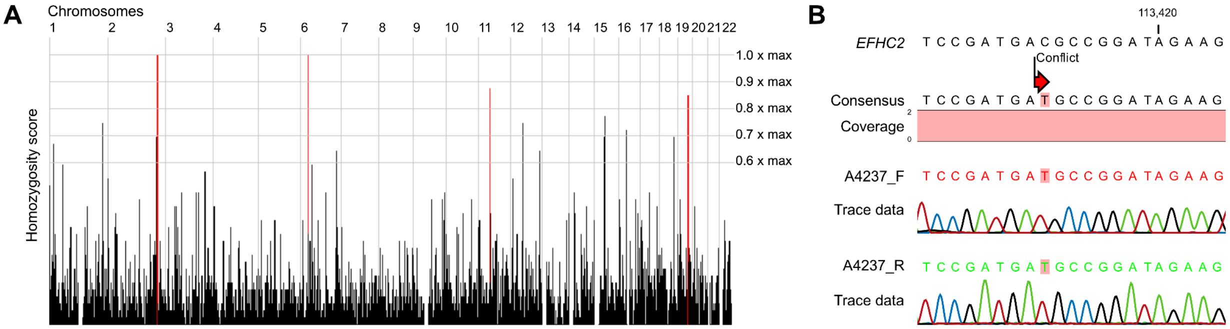

bioRxiv preprint doi: https://doi.org/10.1101/2024.05.26.595818; this version posted May 29, 2024. The copyright holder for this preprint (which was not certified by peer review) is the author/funder, who has granted bioRxiv a license to display the preprint in perpetuity. It is made available under aCC-BY 4.0 International license.

Figure S3.

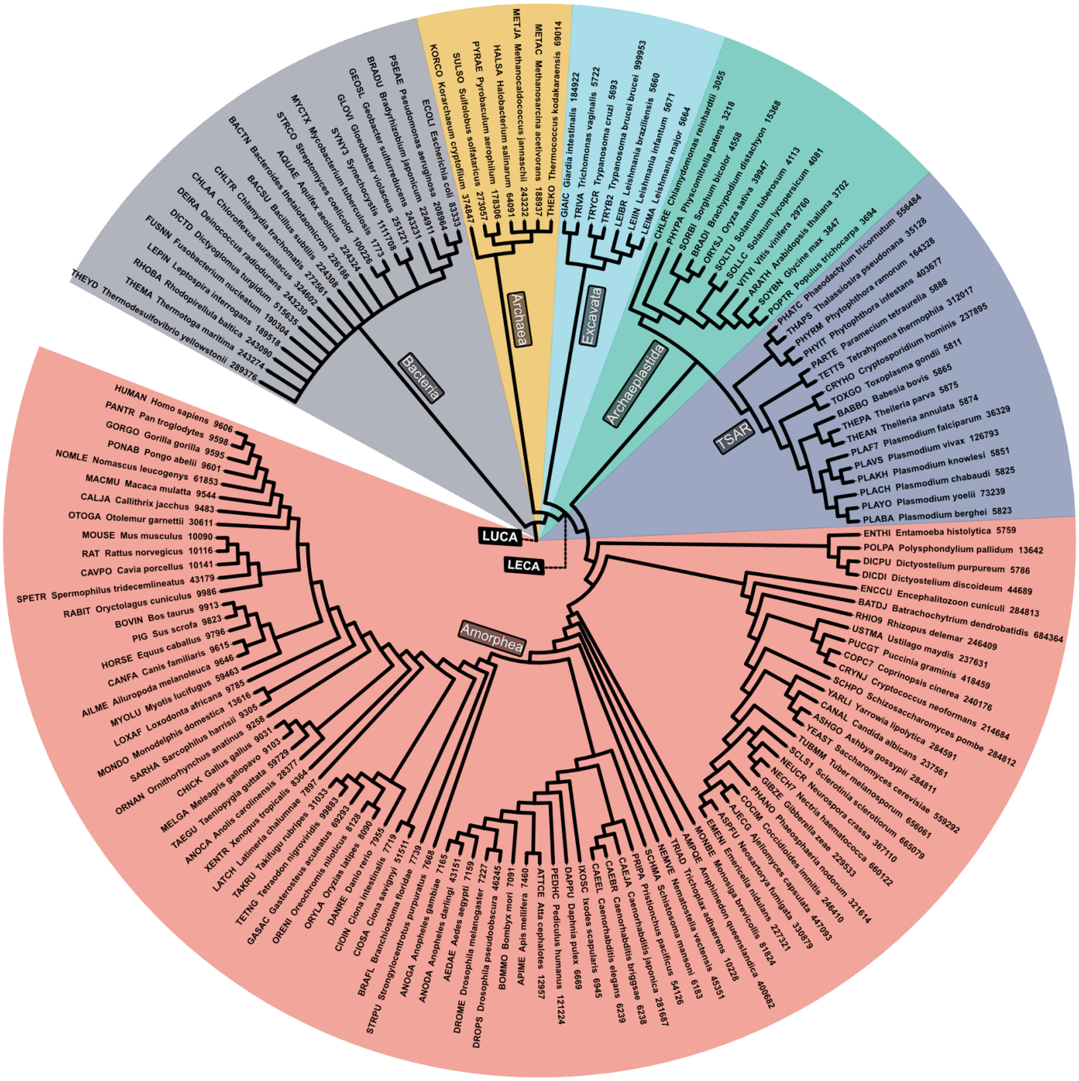

bioRxiv preprint doi: https://doi.org/10.1101/2024.05.26.595818; this version posted May 29, 2024. The copyright holder for this preprint (which was not certified by peer review) is the author/funder, who has granted bioRxiv a license to display the preprint in perpetuity. It is made available under aCC-BY 4.0 International license.

Figure S4.

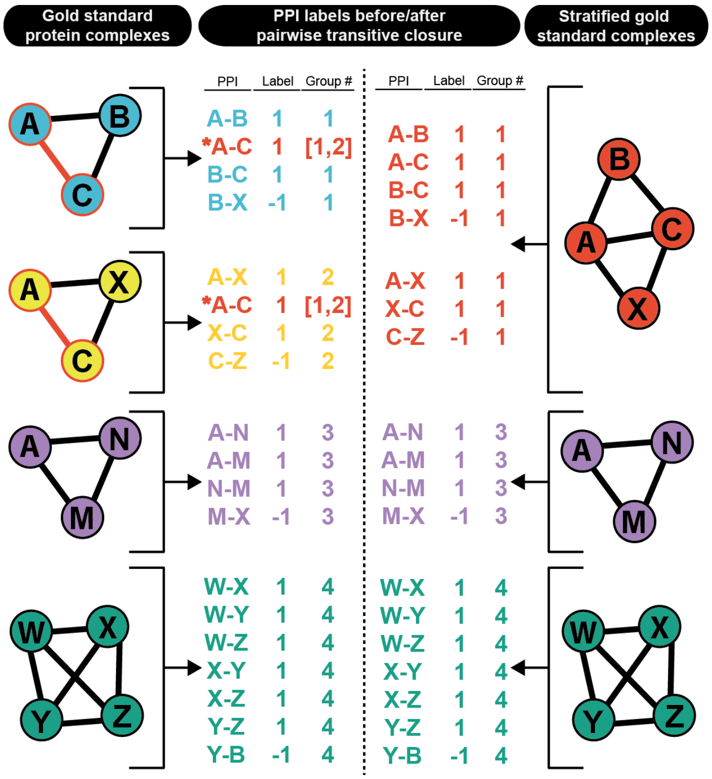

bioRxiv preprint doi: https://doi.org/10.1101/2024.05.26.595818; this version posted May 29, 2024. The copyright holder for this preprint (which was not certified by peer review) is the author/funder, who has granted bioRxiv a license to display the preprint in perpetuity. It is made available under aCC-BY 4.0 International license.

Figure S5.

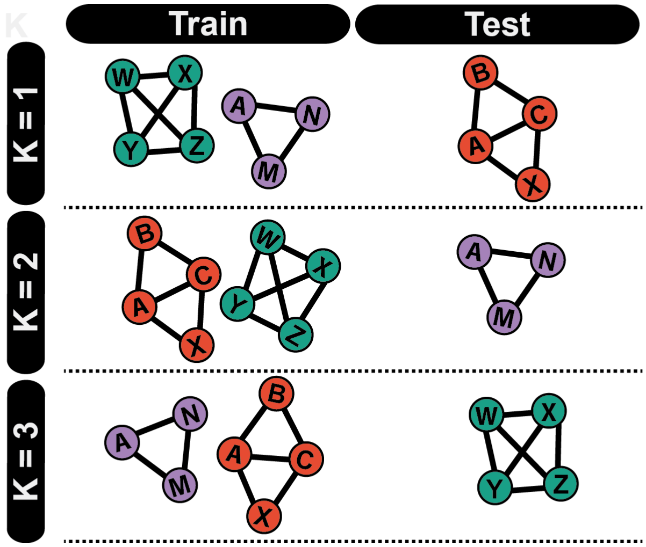

bioRxiv preprint doi: https://doi.org/10.1101/2024.05.26.595818; this version posted May 29, 2024. The copyright holder for this preprint (which was not certified by peer review) is the author/funder, who has granted bioRxiv a license to display the preprint in perpetuity. It is made available under aCC-BY 4.0 International license.

Figure S6.

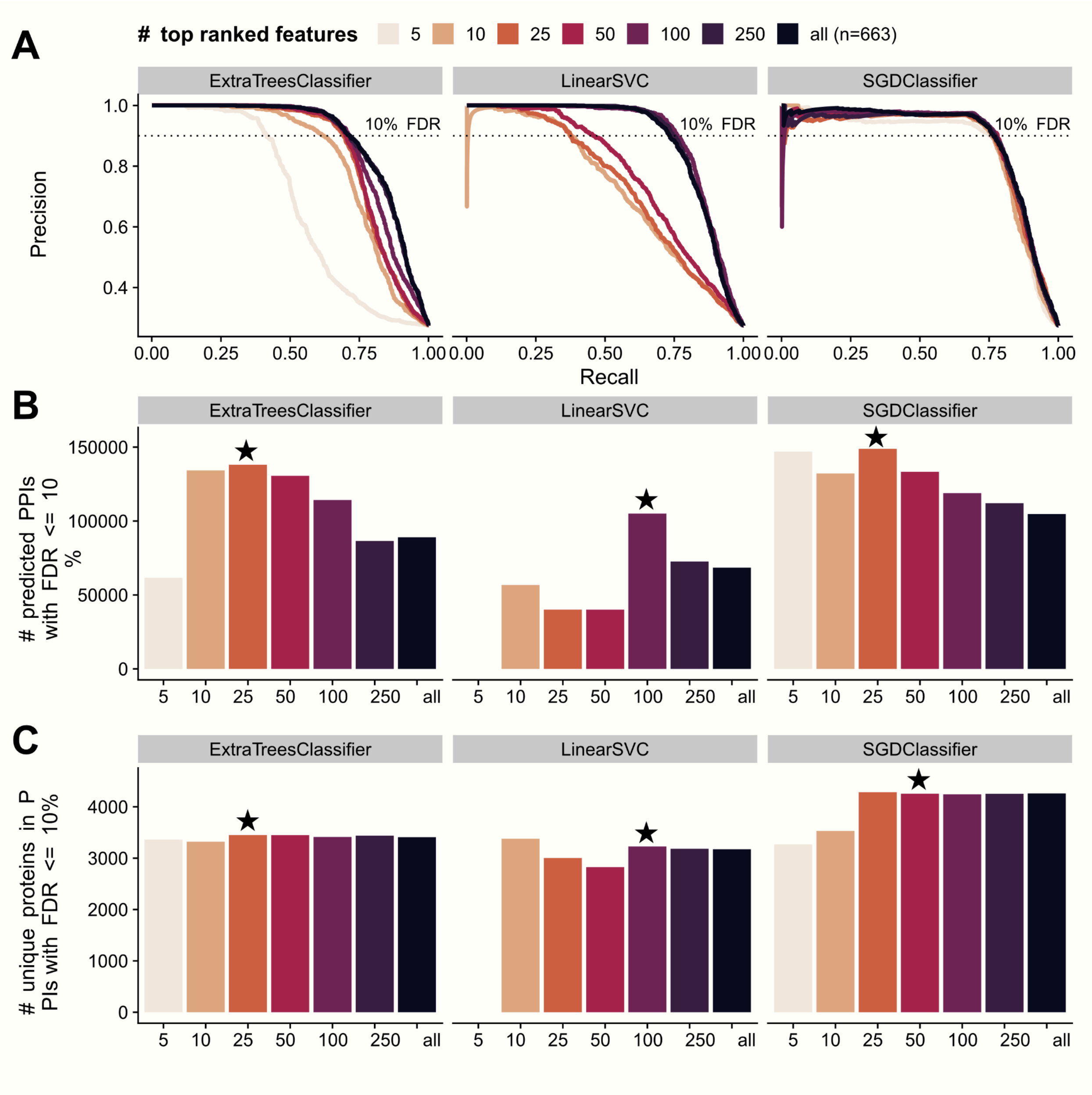

bioRxiv preprint doi: https://doi.org/10.1101/2024.05.26.595818; this version posted May 29, 2024. The copyright holder for this preprint (which was not certified by peer review) is the author/funder, who has granted bioRxiv a license to display the preprint in perpetuity. It is made available under aCC-BY 4.0 International license.

Supplemental Tables

Table S1. 10,092 orthogroups estimated by Dollo parsimony to have been present in LECA. 12MB file, available on Zenodo repository.

bioRxiv preprint doi: https://doi.org/10.1101/2024.05.26.595818; this version posted May 29, 2024. The copyright holder for this preprint (which was not certified by peer review) is the author/funder, who has granted bioRxiv a license to display the preprint in perpetuity. It is made available under aCC-BY 4.0 International license.

Table S2. Summary of biological samples, data sets, software, and algorithms used to derive the conserved eukaryotic interactome.

REAGENT OR RESOURCE	SOURCE	IDENTIFIER
**BIOLOGICAL SAMPLES**		
Euglena gracilis	UTEX Culture Collection of Algae	UTEX 753
Tetrahymena thermophila	Tetrahymena Stock Center	SB175 (SD01508)
Pig trachea (*Sus scrofa*)	Sierra for Medical Science sierra-medical.com	N/A
Diatom (*Phaeodactylum tricornutum*)	UTEX Culture Collection of Algae	UTEX 646
Rotifer (*Brachionus rotundiformis*)	Eberhart lab, UT Austin	S-type
**MASS SPECTROMETRY DATA**		
Euglena gracilis	This work	**PRIDE:** PXD050669
Tetrahymena thermophila	This work	**PRIDE:** PXD050671, PXD050672, PXD050674
Pig (*Sus scrofa*)	This work	**PRIDE:** PXD041980
Diatom (*Phaeodactylum tricornutum*)	This work	**PRIDE:** PXD050670
Rotifer (*Brachionus rotundiformis*)	This work	**PRIDE:** PXD050673
Mouse (*Mus musculus*)	This work	**PRIDE:** PXD041915
	Wan et al., 2015	**PRIDE:** PXD002323
Arabidopsis thaliana	McWhite et al., 2020	**PRIDE:** PXD013264, PXD013321, PXD014617
Broccoli (*Brassica oleracea*)	McWhite et al., 2020	**PRIDE:** PXD013281, PXD013322, PXD013282
Chlamydomonas reinhardtii	McWhite et al., 2020	**PRIDE:** PXD013369, PXD013735
Coconut (*Cocos nucifera*)	McWhite et al., 2020	**PRIDE:** PXD012865
Fern (*Ceratopteris richardii*)	McWhite et al., 2020	**PRIDE:** PXD013320
Hemp (*Cannabis sativa*)	McWhite et al., 2020	**PRIDE:** PXD012969
Maize (*Zea mays*)	McWhite et al., 2020	**PRIDE:** PXD012810
Quinoa (*Chenopodium quinoa*)	McWhite et al., 2020	**PRIDE:** PXD013080
Rice (*Oryza sativa*)	McWhite et al., 2020	**PRIDE:** PXD013213
Selaginella moellendorffii	McWhite et al., 2020	**PRIDE:** PXD013093
Soy (*Glycine max*)	McWhite et al., 2020	**PRIDE:** PXD013198, PXD013704
Tomato (*Solanum lycopersicum*)	McWhite et al., 2020	**PRIDE:** PXD013004

bioRxiv preprint doi: https://doi.org/10.1101/2024.05.26.595818; this version posted May 29, 2024. The copyright holder for this preprint (which was not certified by peer review) is the author/funder, who has granted bioRxiv a license to display the preprint in perpetuity. It is made available under aCC-BY 4.0 International license.

REAGENT OR RESOURCE	SOURCE	IDENTIFIER
Wheat (*Triticum aestivum*)	McWhite et al., 2020	**PRIDE:** PXD013214, PXD013280, PXD013300
Plasmodium berghei	Hillier et al., 2019	**PRIDE:** PXD009039
Plasmodium falciparum	Hillier et al., 2019	**PRIDE:** PXD009039
Plasmodium knowlesi	Hillier et al., 2019	**PRIDE:** PXD009039
Trypanosoma brucei	Crozier et al., 2017	**PRIDE:** PXD005968
Human (*Homo sapiens*)	Wan et al., 2015	**PRIDE:** PXD002322, PXD002328
Worm (*Caenorhabditis elegans*)	Wan et al., 2015	**PRIDE:** PXD002319
Slime mold (*Dictyostelium discoideum*)	Wan et al., 2015	**PRIDE:** PXD002320
Fly (*Drosophila melanogaster*)	Wan et al., 2015	**PRIDE:** PXD002321
Sea anemone (*Nematostella vectensis*)	Wan et al., 2015	**PRIDE:** PXD002324
Sea urchin (*Strongylocentrotus purpuratus*)	Wan et al., 2015	**PRIDE:** PXD002325
African clawed frog (*Xenopus laevis*)	Drew et al., 2020; Wan et al., 2015	**PRIDE:** PXD017650, PXD017659; PXD002326
Yeast (*Saccharomyces cerevisiae*)	Wan et al., 2015	**PRIDE:** PXD002327
**SOFTWARE AND ALGORITHMS**		
Orthogroup inference	eggNOG v2.0.5	https://github.com/eggnogdb/eggnog-mapper/releases
Reference database construction	This paper	https://github.com/marcottelab/leca-proteomics/scripts/concat_ortho_proteins.py
Peptide identification	MSblender	https://github.com/marcottelab/MSblender
Ancestral genome inference	Count	http://www.iro.umontreal.ca/~csuros/gene_content/count.html
Feature extraction (CFMS data)	McWhite et al., 2020	https://github.com/marcottelab/protein_complex_maps/tree/master/protein_complex_maps/features/ExtractFeatures/canned_scripts/extract_features.py
Feature integration (APMS data)	hu.MAP 2.0	http://humap2.proteincomplexes.org/static/downloads/humap2/
	This paper	https://github.com/marcottelab/leca-proteomics/notebooks/map_entrez_to_eggnog.ipynb
Pairwise protein interaction labels	This paper	https://github.com/marcottelab/leca-proteomics/scripts/label_featmat.py
Model optimization	TPOT 0.11.7	http://epistasislab.github.io/tpot/
	This paper	https://github.com/marcottelab/leca-proteomics/scripts/run_tpot.py

bioRxiv preprint doi: https://doi.org/10.1101/2024.05.26.595818; this version posted May 29, 2024. The copyright holder for this preprint (which was not certified by peer review) is the author/funder, who has granted bioRxiv a license to display the preprint in perpetuity. It is made available under aCC-BY 4.0 International license.

REAGENT OR RESOURCE	SOURCE	IDENTIFIER
Feature/model selection	This paper	https://github.com/marcottelab/leca-proteomics/scripts/select_features.py
Final model generation/assessment	This paper	https://github.com/marcottelab/leca-proteomics/scripts/predict_ppis.py
Protein complex detection	This paper	https://github.com/marcottelab/leca-proteomics/scripts/detect_communities.py

bioRxiv preprint doi: https://doi.org/10.1101/2024.05.26.595818; this version posted May 29, 2024. The copyright holder for this preprint (which was not certified by peer review) is the author/funder, who has granted bioRxiv a license to display the preprint in perpetuity. It is made available under aCC-BY 4.0 International license.

Table S3. 3,193 LECA orthogroups organized hierarchically into 2,013 protein assemblies. 1.3 MB file, available on Zenodo repository.

bioRxiv preprint doi: https://doi.org/10.1101/2024.05.26.595818; this version posted May 29, 2024. The copyright holder for this preprint (which was not certified by peer review) is the author/funder, who has granted bioRxiv a license to display the preprint in perpetuity. It is made available under aCC-BY 4.0 International license.

Table S4. Reference proteomes sourced for co-fractionation mass spectrometry data processing.

Species Code	Species Name	Proteome Source	Date Accessed
ARATH	*Arabidopsis thaliana* (Mouse-ear cress)	Proteome downloaded from https://www.uniprot.org/proteomes/UP000006548	2/5/2021
BRAOL	*Brassica oleracea* (broccoli)	Proteome downloaded from https://www.uniprot.org/proteomes/UP000032141	2/5/2021
BRART	*Brachionus* rotundiformis (rotifer)	Transcriptome downloaded from https://www.ncbi.nlm.nih.gov/Traces/wgs/GINZ01?display=contigs	2/5/2021
CAEEL	*Caenorhabditis elegans*	Proteome downloaded from https://www.uniprot.org/proteomes/UP000001940	2/5/2021
CANSA	*Cannabis sativa* (hemp)	Proteome downloaded from http://genome.ccbr.utoronto.ca/downloads.html	2/5/2021
CERRI	*Ceratopteris richardii* (fern)	Proteome downloaded from https://zenodo.org/record/3467771#.YB2hB-hKguU	2/5/2021
CHEQI	*Chenopodium quinoa* (quinoa)	Proteome downloaded from https://genome.jgi.doe.gov/portal/pages/dynamicOrganismDownload.jsf?organism=Cquinoa	2/5/2021
CHLRE	*Chlamydomonas reinhardtii* (*Chlamydomonas smithii*)	Proteome downloaded from https://www.uniprot.org/proteomes/UP000006906	2/5/2021
COCNU	*Cocos nucifera* (coconut)	Proteome downloaded from https://www.ncbi.nlm.nih.gov/assembly/GCA_008124465.1/	2/5/2021
DICDI	*Dictyostelium discoideum* (Slime mold)	Proteome downloaded from https://www.uniprot.org/proteomes/UP000002195	2/5/2021
DROME	*Drosophila melanogaster* (Fruit fly)	Proteome downloaded from https://www.uniprot.org/proteomes/UP000000803	2/5/2021
EUGGR	*Euglena gracilis* (algae)	Proteome downloaded from ftp://ftp.pride.ebi.ac.uk/pride/data/archive/2019/01/PXD009998	2/5/2021
HUMAN	*Homo sapiens* (Human)	Proteome downloaded from https://www.uniprot.org/proteomes/UP000005640	2/5/2021
MAIZE	*Zea mays* (Maize)	Proteome downloaded from https://www.uniprot.org/proteomes/UP000007305	2/5/2021
MOUSE	*Mus musculus* (Mouse)	Proteome downloaded from https://www.uniprot.org/proteomes/UP000000589	2/5/2021
NEMVE	*Nematostella vectensis* (Starlet sea anemone)	Proteome downloaded from https://www.uniprot.org/proteomes/UP000001593	2/5/2021
ORYSJ	*Oryza sativa* subsp. *japonica* (Rice)	Proteome downloaded from https://www.uniprot.org/proteomes/UP000059680	2/5/2021

bioRxiv preprint doi: https://doi.org/10.1101/2024.05.26.595818; this version posted May 29, 2024. The copyright holder for this preprint (which was not certified by peer review) is the author/funder, who has granted bioRxiv a license to display the preprint in perpetuity. It is made available under aCC-BY 4.0 International license.

Species Code	Species Name	Proteome Source	Date Accessed
PHATC	*Phaeodactylum tricornutum* (diatom, strain CCAP 1055/1)	Proteome downloaded from https://www.uniprot.org/proteomes/UP000000759	2/5/2021
PIG	*Sus scrofa* (wild boar)	Proteome downloaded from https://www.uniprot.org/proteomes/UP000008227	2/5/2021
PLABA	*Plasmodium berghei* (strain Anka)	Proteome downloaded from https://www.uniprot.org/proteomes/UP000074855	2/8/2021
PLAF7	*Plasmodium falciparum* (isolate 3D7)	Proteome downloaded from https://www.uniprot.org/proteomes/UP000001450	2/5/2021
PLAKH	*Plasmodium knowlesi* (strain H)	Proteome downloaded from https://www.uniprot.org/proteomes/UP000031513	2/8/2021
SELML	*Selaginella moellendorffii* (spikemoss)	Proteome downloaded from https://www.uniprot.org/proteomes/UP000001514	2/5/2021
SOLLC	*Solanum lycopersicum* (tomato)	Proteome downloaded from https://www.uniprot.org/proteomes/UP000004994	2/5/2021
SOYBN	*Glycine max* (soybean)	Proteome downloaded from https://www.uniprot.org/proteomes/UP000008827	2/5/2021
STRPU	*Strongylocentrotus purpuratus* (purple sea urchin)	Proteome downloaded from https://www.uniprot.org/proteomes/UP000007110	2/5/2021
TETTS	*Tetrahymena thermophila* (ciliate, strain SB210)	Proteome downloaded from https://www.uniprot.org/proteomes/UP000009168	2/5/2021
TRYB2	*Trypanosoma brucei brucei* (strain 927/4 GUTat10.1)	Proteome downloaded from https://www.uniprot.org/proteomes/UP000008524	2/22/2021
WHEAT	*Triticum aestivum* (wheat)	Proteome downloaded from https://www.uniprot.org/proteomes/UP000019116	2/5/2021
XENLA	*Xenopus laevis* (African clawed frog)	Proteome downloaded from https://www.uniprot.org/proteomes/UP000186698	2/5/2021
YEAST	*Saccharomyces cerevisiae* (strain ATCC 204508 / S288c) (Baker's yeast)	Proteome downloaded from https://www.uniprot.org/proteomes/UP000002311	2/5/2021

bioRxiv preprint doi: https://doi.org/10.1101/2024.05.26.595818; this version posted May 29, 2024. The copyright holder for this preprint (which was not certified by peer review) is the author/funder, who has granted bioRxiv a license to display the preprint in perpetuity. It is made available under aCC-BY 4.0 International license.

Table S5. Summary of the top scoring algorithms, parameters and pre-processing steps found by TPOT. Models marked with a star (★) were selected for further evaluation.

Model	Pre-processing Steps	Parameters	TPOT CV Score	True Test Score
★ LinearSVC	RobustScaler(), ZeroCount(), VarianceThreshold (threshold=0.01)	C=0.01, dual=False, loss="squared_hinge", penalty="l1", tol=0.01	0.869	0.878
ExtraTreesClassifier	OneHotEncoder (minimum_fraction= 0.15, sparse=False, threshold=10)	bootstrap=False, criterion="gini", max_features=0.6000000000000001, min_samples_leaf=11, min_samples_split=12, n_estimators=100	0.911	0.848
★ ExtraTreesClassifier	None	bootstrap=True, criterion="gini", max_features=0.6000000000000001, min_samples_leaf=15, min_samples_split=2, n_estimators=100	0.87	0.891
ExtraTreesClassifier	Normalizer (norm="max")	bootstrap=False, criterion="entropy", max_features=0.35000000000000003, min_samples_leaf=15, min_samples_split=3, n_estimators=100	0.867	0.854
★ SGDClassifier	Normalizer (norm="l1"), StandardScaler()	alpha=0.01, eta0=0.01, fit_intercept=False, l1_ratio=1.0, learning_rate="invscaling", loss="modified_huber", penalty="elasticnet", power_t=0.5	0.87	0.897
